# Human LACC1 increases innate receptor-induced responses and a *LACC1* disease-risk variant modulates these outcomes

**DOI:** 10.1038/ncomms15614

**Published:** 2017-06-08

**Authors:** Amit Lahiri, Matija Hedl, Jie Yan, Clara Abraham

**Affiliations:** 1 Department of Internal Medicine, Section of Digestive Diseases, Yale University, New Haven, Connecticut 06510, USA

## Abstract

Functional consequences for most inflammatory disease-associated loci are incompletely defined, including in the *LACC1* (*C13orf31*) region. Here we show that human peripheral and intestinal myeloid-derived cells express laccase domain-containing 1 (LACC1); LACC1 is expressed in both the cytoplasm and mitochondria. Upon NOD2 stimulation of human macrophages, LACC1 associates with the NOD2-signalling complex, and is critical for optimal NOD2-induced signalling, mitochondrial ROS (mtROS) production, cytokine secretion and bacterial clearance. LACC1 constitutively associates with succinate dehydrogenase (SDH) subunit A, and amplifies pattern recognition receptor (PRR)-induced SDH activity, an important contributor to mtROS production. Relative to LACC1 Ile254, cells transfected with Crohn's disease-risk LACC1 Val254 or LACC1 with mutations of the nearby histidines (249,250) have reduced PRR-induced outcomes. Relative to LACC1 Ile254 carriers, Val254 disease-risk carrier macrophages demonstrate decreased PRR-induced mtROS, signalling, cytokine secretion and bacterial clearance. Therefore, LACC1 is critical for amplifying PRR-induced outcomes, an effect that is attenuated by the *LACC1* disease-risk variant.

## Abstract (web summary)

*LACC1* genetic variants are associated with Crohn's disease, leprosy and arthritis. Here the authors show that LACC1 is needed for optimal innate receptor-induced signalling, mitochondrial ROS production and microbial clearance, effects that are reduced by a Crohn's disease-risk variant in *LACC1*.

Inflammatory bowel disease (IBD) is characterized by dysregulated host–microbial interactions and cytokine production^1^. Microbial recognition and responses are mediated by host pattern recognition receptors (PRRs). Either loss-of-function or gain-of-function in PRR-mediated signalling and downstream outcomes can be associated with intestinal inflammation^2^, highlighting the balance in regulation of PRR-mediated outcomes as critical in intestinal tissues. An important role for host–microbial interactions is further highlighted by Crohn's disease (CD)-associated loss-of-function polymorphisms in *NOD2* (refs 1, 2; encoding NOD2, a PRR that recognizes bacterial peptidoglycan) as well as in additional pathways regulating microbial clearance mechanisms, such as autophagy^3^ and NADPH complex-mediated generation of reactive oxygen species (ROS)^4^,^5^. Despite the success in identification of IBD-associated loci^6^, altered functions for most of the variants in these loci are unknown or not well defined. One such region is at chromosome 13q14 (*C13orf31)*, encompassing the *LACC1* gene^6^.

Polymorphisms in *LACC1* are associated with CD, ankylosing spondylitis, leprosy and juvenile idiopathic arthritis^6^,^7^,^8^,^9^,^10^,^11^. The rs3764147 G risk allele results in an amino-acid change from isoleucine to valine at position 254 in laccase domain-containing protein 1 (LACC1). LACC1 homologues (for example, cytotoxic necrotizing factor (CNF)) in bacteria, fungi and plants can function as virulence factors and in apoptosis prevention^12^,^13^. Laccase domain-containing proteins exhibit enzymatic activity, including oxidase/reductase activity, against various substrates such as polyphenols, aromatic amines and select inorganic ions^13^. Polyphenols mediate a number of host-protective functions^14^,^15^,^16^, and, as such, laccase domain-containing proteins have also been shown to confer protection to various organisms in which they are expressed^13^. To date, laccase domain-containing proteins have been a particular focus for industry, including the textile, environmental, pharmaceutical and food industries^17^. Given the importance of host–microbial interactions in intestinal immune homeostasis, and the critical role of PRRs in recognizing and responding to microbes, we questioned whether LACC1 regulates PRR-initiated outcomes in human myeloid-derived cells. We further questioned whether the IBD risk polymorphism in *LACC1* resulting in an Ile254Val amino-acid change modulates PRR outcomes in a genotype-dependent manner.

We identify that LACC1 is expressed in both peripheral and intestinal human myeloid-derived cells and is required for optimal PRR-induced mitochondrial ROS (mtROS) and ROS production, MAPK and NFκB pathway activation, cytokine secretion and intracellular bacterial clearance in primary human monocyte-derived macrophages (MDMs). We define mechanisms and structural regions in LACC1 regulating these LACC1-dependent functions, as well as a reduction in each of these functions in MDMs from LACC1 Val254 (rs3764147 G) IBD risk, relative to Ile254 non-risk carriers. Taken together, we identify roles for human LACC1, and establish loss-of-function consequences for the LACC1 Val254 IBD risk variant, thereby resulting in decreased amplification of PRR-induced mtROS, signalling, cytokines and bacterial clearance.

## Results

### LACC1 Val254 risk MDMs show reduced PRR-induced cytokines

**Figure 1:**
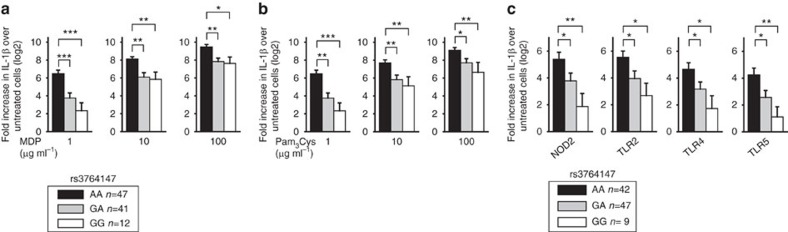
Myeloid cells from LACC1 Val254 disease-risk carriers demonstrate decreased PRR-induced cytokine secretion. Human MDMs (*n*=100) were treated for 24 h with (**a**) 1, 10 or 100 μg ml^−1^ MDP, or (**b**) 1, 10 or 100 μg ml^−1^ Pam_3_Cys. (**c**) Human MDDCs (*n*=98) were treated for 24 h with 1 μg ml^−1^ MDP (NOD2 ligand), 1 μg ml^−1^ Pam_3_Cys (TLR2 ligand), 0.01 μg ml^−1^ lipid A (TLR4 ligand) or 0.5 ng ml^−1^ flagellin (TLR5 ligand) for 24 h. Shown is fold IL-1β-secreted protein induction (log_2_-transformed) upon PRR stimulation stratified on rs3764147 genotype. Error bars depict s.e.m. *P<0.05; **P<0.01; ***P<0.001; determined by 2-tailed *t*-test.

Given the importance of regulating PRR-initiated outcomes in intestinal immune homeostasis and the dysregulation in PRR pathway outcomes that can be observed in IBD^1^, we questioned whether the rs3764147 genotype in human *LACC1* modulates PRR-initiated outcomes in human MDMs. As *NOD2* is associated to CD, we treated MDMs from 100 healthy individuals with muramyl dipeptide (MDP), the minimal bacterial peptidoglycan component activating NOD2 (refs 18, 19). We examined interleukin (IL)-1β-secreted protein, which amplifies PRR-mediated signalling and cytokine secretion in MDMs^20^ and is elevated in tissues from IBD patients^21^. We normalized IL-1β protein secretion to untreated cells and log2-transformed the data. LACC1 Val254 disease-risk carriers (rs3764147 G carriers) secreted less IL-1β protein relative to Ile254 carriers (rs3764147 AA; Fig. 1a). This was most pronounced at low MDP doses (Fig. 1a). The pro-inflammatory cytokine tumour-necrosis factor (TNF; Supplementary Fig. 1A) was similarly regulated. Anti-inflammatory cytokines can sometimes be regulated in a reciprocal manner; however, the anti-inflammatory cytokine IL-10 was regulated similarly to the pro-inflammatory cytokines (Supplementary Fig. 1D). Moreover, less IL-1β (Fig. 1b), TNF (Supplementary Fig. 1B) and IL-10 (Supplementary Fig. 1E) secretion was observed in MDMs from LACC1 Val254 carriers upon stimulation with the TLR2 ligand, Pam_3_Cys.

Microbial products activate multiple PRRs, and in addition to macrophages, can activate PRRs on dendritic cells. In a separate cohort of 98 healthy individuals, LACC1 Val254 risk carrier monocyte-derived dendritic cells (MDDCs) secreted lower levels of IL-1β, TNF and IL-10 protein upon stimulation of NOD2, TLR2, TLR4 and TLR5 (Fig. 1c and Supplementary Fig. 1C,F). Therefore, myeloid cells from LACC1 Val254 risk carriers secrete less PRR-induced cytokines than LACC1 Ile254 carriers.

### LACC1 expression increases with PRR stimulation

**Figure 2:**
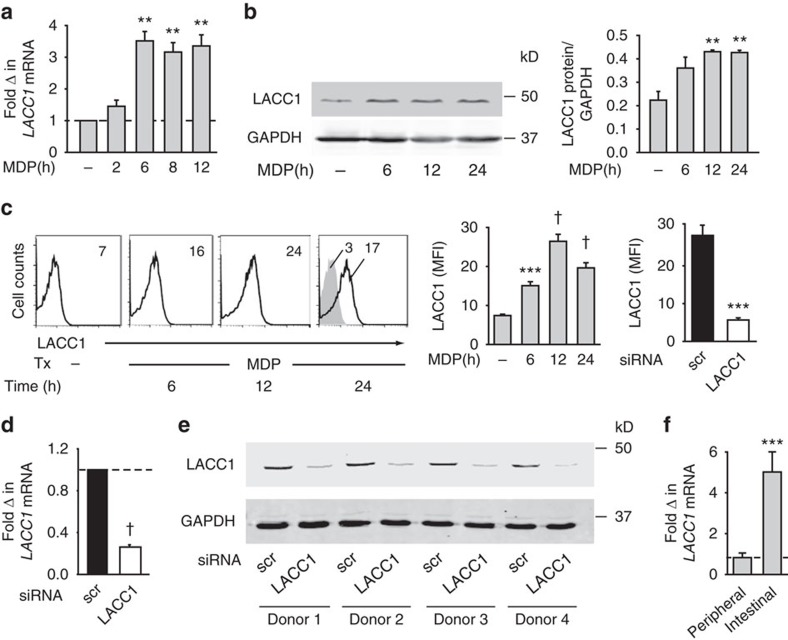
LACC1 is expressed in human MDMs and intestinal myeloid cells. Human MDMs were treated with 100 μg ml^−1^ MDP for the indicated times and assessed for: (**a**) *LACC1* mRNA expression (*n*=4; with similar results for an additional *n*=4), (**b**) LACC1 protein expression by western blot with GAPDH as a loading control along with a summary graph of densitometry in which samples are normalized to GAPDH (*n*=4) and (**c**; Left) LACC1 protein expression by flow cytometry. Representative and summarized flow cytometry with the mean fluorescent intensity (MFI) values shown (*n*=7). Isotype (grey shading). Representation of population gated is shown in Supplementary Fig. 2A. (Right) MDMs were transfected with scrambled or LACC1 siRNA and then treated with MDP for 12 h. Summarized graph for MFI of samples assessed by flow cytometry+s.e.m. (*n*=7 donors). (**d**,**e**) MDMs were transfected with scrambled or LACC1 siRNA and (**d**) *LACC1* mRNA expression+s.e.m. (*n*=4), and (**e**) representative western blot for LACC1 expression is shown for 4 of 20 donors. (**f**) *LACC1* mRNA expression was assessed in intestinal (*n*=7) and peripheral (*n*=7) myeloid-derived cells and normalized to *CD11c*. Mean+s.e.m. Scr, scrambled; tx, treatment. **P<0.01; ***P<0.001; ^†^*P*<1 × 10^−4^; determined by 2-tailed *t*-test.

We next assessed LACC1 expression in human MDMs. Upon NOD2 stimulation of human MDMs, *LACC1* mRNA expression increased within 6 h, with elevated expression persisting over 12 h (Fig. 2a). LACC1 protein expression similarly increased with NOD2 stimulation as assessed by both western blot (Fig. 2b) and flow cytometry (Fig. 2c). To confirm the specificity of the antibody, we utilized short interfering RNA (siRNA) to LACC1 and observed significant attenuation of *LACC1* mRNA (Fig. 2d) and protein (Fig. 2c,e) expression. Finally, we examined human intestinal myeloid-derived cells, given the association of polymorphisms in *LACC1* with IBD. LACC1 was expressed in these tissue-relevant cells to an even higher level than in peripheral myeloid-derived cells (Fig. 2f), consistent with the ongoing PRR ligand exposure in this environment and the ability of PRR stimulation to increase LACC1 expression. Therefore, LACC1 is expressed in both human intestinal and peripheral myeloid-derived cells, with expression increasing upon PRR stimulation.

### LACC1 contributes to PRR-induced cytokines

**Figure 3:**
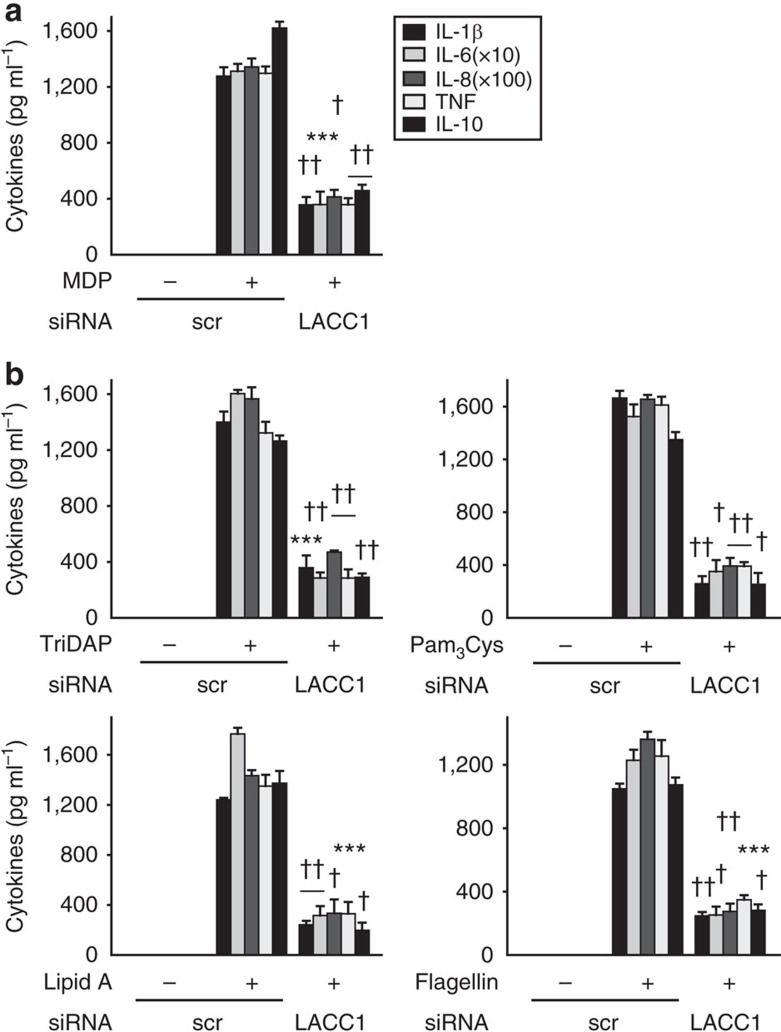
LACC1 amplifies PRR-induced cytokines. MDMs were transfected with scrambled or LACC1 siRNA, and then treated for 24 h with (**a**) 100 μg ml^−1^ MDP (*n*=4; similar results were observed in an additional *n*=18), or (**b**) 100 μg ml^−1^ TriDAP, 10 μg ml^−1^ Pam_3_Cys, 0.1 μg ml^−1^ lipid A or 5 ng ml^−1^ flagellin (*n*=4; similar results were observed in an additional *n*=8). Cytokine secretion+s.e.m. Scr, scrambled. ***P<0.001; ^†^*P*<1 × 10^−4^; ^††^*P*<1 × 10^−5^; determined by 2-tailed *t*-test.

We next sought to clearly establish that LACC1 is required for regulating PRR-induced cytokines. Upon effective knockdown of LACC1 (Fig. 2c–e), NOD2-induced pro-inflammatory and anti-inflammatory cytokine secretion was reduced (Fig. 3a). The cells were viable (Supplementary Fig. 2B), and anti-inflammatory cytokine secretion through dectin ligands was not affected with LACC1 knockdown (Supplementary Fig. 2C). We confirmed these results with three additional siRNAs to LACC1 (Supplementary Fig. 2D,E). Importantly, we determined that LACC1 was required for optimal cytokine secretion upon stimulation of multiple PRRs (Fig. 3b). We ensured that with LACC1 knockdown, the expression of each of these PRRs was not altered (Supplementary Fig. 3A). *LACC1* region polymorphisms are associated with leprosy^9^, and we found that LACC1 was also required for optimal cytokine secretion by whole-cell *Mycobacterium leprae* lysate and its cytosolic and membrane fractions (Supplementary Fig. 4A). The dependency on LACC1 extended to additional mycobacteria, in particular to *M. tuberculosis* components (Supplementary Fig. 4B). Of note is that we did not detect mycobacteria (16s rDNA for *M. tuberculosis, M. leprae* and *M. avium,* subspecies *paratuberculosis*) in whole blood from 200 healthy controls (*n*=85, 76, 39 Ile254 homozygotes, Ile/Val254 heterozygotes and Val254 homozygotes, respectively) or 40 CD patients (*n*=18, 16 and 6 Ile254 homozygotes, Ile/Val254 heterozygotes and Val254 homozygotes, respectively). Therefore, LACC1 is required for optimal cytokine secretion by multiple PRRs and mycobacterial components, thereby highlighting LACC1 as a shared pathway mediating responses to these microbes.

### LACC1 increases PRR-induced MAPK and NFκB activation

**Figure 4:**
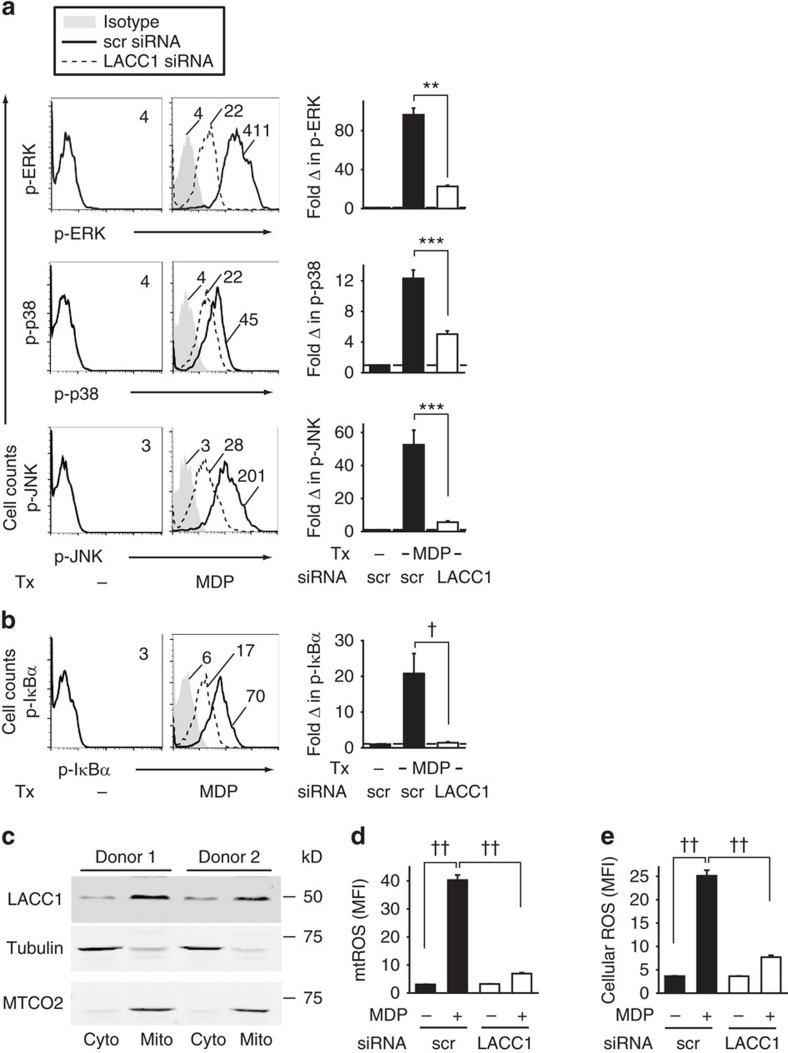
LACC1 is required for optimal PRR-induced signalling and mtROS production. (**a**,**b**) MDMs (*n*=4) were transfected with scrambled or LACC1 siRNA, and then treated for 15 min with 100 μg ml^−1^ MDP. Left: representative flow cytometry plots with MFI values for (**a**) phospho-ERK, phospho-p38 and phospho-JNK, and (**b**) phospho-IκBα. Right: fold phospho-protein induction normalized to untreated cells (represented by the dotted line at 1)+s.e.m. Similar results were observed for an additional *n*=8. In a subset of individuals for **a**,**b** we simultaneously confirmed reduced LACC1 expression with LACC1 siRNA by western blot (Supplementary Fig. 5A) and flow cytometry. (**c**) MDMs were fractionated into cytoplasmic (cyto) and mitochondrial (mito) compartments and assessed for LACC1 expression. Shown is a representative western blot for LACC1 in two of six individuals. Tubulin and MTCO2 were used as cytoplasmic and mitochondrial loading controls, respectively. (**d**,**e**) MDMs were transfected with scrambled or LACC1 siRNA, and then treated with 100 μg ml^−1^ MDP for 6 h and assessed for: (**d**) mtROS (*n*=4) and (**e**) cellular ROS (*n*=4). Shown is mean+s.e.m. Similar results were seen in an additional *n*=8 donors. In a subset of individuals for **d**,**e** we simultaneously confirmed reduced LACC1 expression with LACC1 siRNA by western blot (Supplementary Fig. 7B) and flow cytometry. scr, scrambled; Tx, treatment. **P<0.01; ***P<0.001; ^†^*P*<1 × 10^−4^; ^††^*P*<1 × 10^−5^; determined by 2-tailed *t*-test.

We next sought to determine which signalling pathways LACC1 was amplifying during PRR activation. MAPKs and NFκB pathways are activated downstream of NOD2 (refs 20, 22, 23), and contribute to cytokine secretion^23^,^24^. Silencing of LACC1 during NOD2 stimulation of MDMs led to reduced ERK, p38 and JNK activation (Fig. 4a; total protein levels were unchanged; Supplementary Fig. 5B), and NFκB activation as assessed by phospho-flow cytometry (Fig. 4b) and western blot (Supplementary Fig. 5C).

### LACC1 modulates a broad range of NOD2-induced transcripts

As LACC1 regulates both NOD2-induced MAPK and NFκB pathways, and these signalling pathways in turn regulate a broad range of NOD2-induced transcripts, we postulated that in addition to its regulation of PRR-induced cytokines, LACC1 would regulate a broad spectrum of NOD2-induced transcripts. We focused on the NOD2-upregulated transcripts identified through microarray in ref. 25, and found that the upregulation of these transcripts was impaired with LACC1 knockdown (Supplementary Fig. 6A,C). To demonstrate the MAPK/NFκB signalling dependency of these transcripts, we examined these same transcripts upon NOD2 stimulation while inhibiting MAPK and NFκB pathways and found that NOD2-induced transcript upregulation was similarly impaired (Supplementary Fig. 6A,C). We ensured that the cells were viable under these conditions (Supplementary Fig. 6B). As a control for specificity of LACC1 knockdown effects, we examined dectin-induced transcripts, as we had found that dectin-induced IL-10 did not depend on LACC1 (Supplementary Fig. 2C). A subset of NOD2-induced transcripts was not upregulated with dectin stimulation (Supplementary Fig. 6A). However, we selected transcripts previously reported to be regulated by dectin^26^, and found that dectin-induced transcripts were not significantly altered with LACC1 knockdown (Supplementary Fig. 6D), including transcripts that were also induced upon NOD2 stimulation (Supplementary Fig. 6C). Therefore, consistent with the LACC1 regulation of broad NOD2-induced signalling pathways, LACC1 modulates a broad range of NOD2-induced transcripts.

### LACC1 increases NOD2-induced mitochondrial-derived ROS

We next questioned the mechanisms through which LACC1 regulates PRR-induced signalling and cytokines. CNF1, a bacterial homologue of LACC1, can prevent apoptosis by altering mitochondrial function in host cells^12^. Mitochondria can contribute to PRR-induced outcomes through various mechanisms, including through production of mtROS^27^,^28^. We found that LACC1 was expressed in both the mitochondrial and cytosolic cellular fractions (Fig. 4c). The isolated mitochondrial fraction did not demonstrate expression of the ER marker protein disulfide isomerase (PDI), whereas PDI was present in whole lysate (Supplementary Fig. 7A). LACC1 homologues in bacteria and fungi exhibit oxidase/reductase activity against polyphenols^13^; polyphenol end products can modulate ROS production^14^. LACC1 knockdown reduced mtROS upon NOD2 stimulation in MDMs (Fig. 4d). Total cellular ROS was similarly reduced (Fig. 4e). MtROS is known to contribute to PRR-induced cytokines through various mechanisms^27^,^28^. We confirmed that mtROS contributed to NOD2-induced cytokine secretion by two independent approaches, through use of the mtROS scavenger Mito-Tempo and knockdown of the voltage-dependent anion channel 3 (VDAC3) (Supplementary Fig. 7C), which regulates mtROS. In both cases, we observed the expected decrease in NOD2-induced mtROS and cellular ROS (Supplementary Fig. 7D,E), which was in turn accompanied by a decrease in NOD2-induced signalling (Supplementary Fig. 7F,G), and cytokines (Supplementary Fig. 7H,I). Cell viability was intact under both these conditions (Supplementary Fig. 7J,K). Furthermore, LACC1 was required for optimal mtROS and ROS induction upon stimulation through a range of PRRs (Supplementary Fig. 8A). We ensured that the commonly utilized ligands to activate these PRRs were, in fact, signalling through their respective PRRs as assessed by lack of induction of mtROS, ROS and cytokines upon knockdown of the appropriate PRR (Supplementary Fig. 8B,C). Therefore, LACC1 is required for optimal PRR-induced mtROS, which, in turn, regulates PRR-induced cytokine secretion in MDMs.

### NOD2-induced LACC1 expression regulates outcomes

As LACC1 expression in primary human MDMs increased with NOD2 stimulation (Fig. 2), we sought to establish that this increased LACC1 expression was clearly modulating the LACC1-dependent outcomes we had identified upon NOD2 stimulation. To address this, we progressively reduced the elevated LACC1 expression under NOD2-stimulated conditions (Supplementary Fig. 9A), and observed a LACC1 dose-dependent contribution to the increased mtROS (Supplementary Fig. 9B), ROS (Supplementary Fig. 9B), cytokines (Supplementary Fig. 9C) and transcripts (Supplementary Fig. 9D) with NOD2 stimulation. Moreover, the downstream outcomes observed with MDP treatment were clearly dependent on the NOD2 pathway, as knockdown of both NOD2 (Supplementary Fig. 3) and the NOD2 adaptor molecule RIP2 (Supplementary Fig. 10A) led to a loss of the increased LACC1 expression, mtROS, ROS, signalling and cytokines observed with MDP treatment (Supplementary Fig. 10B–E).

### LACC1 Val254 or His249,250Ala reduces PRR-induced outcomes

**Figure 5:**
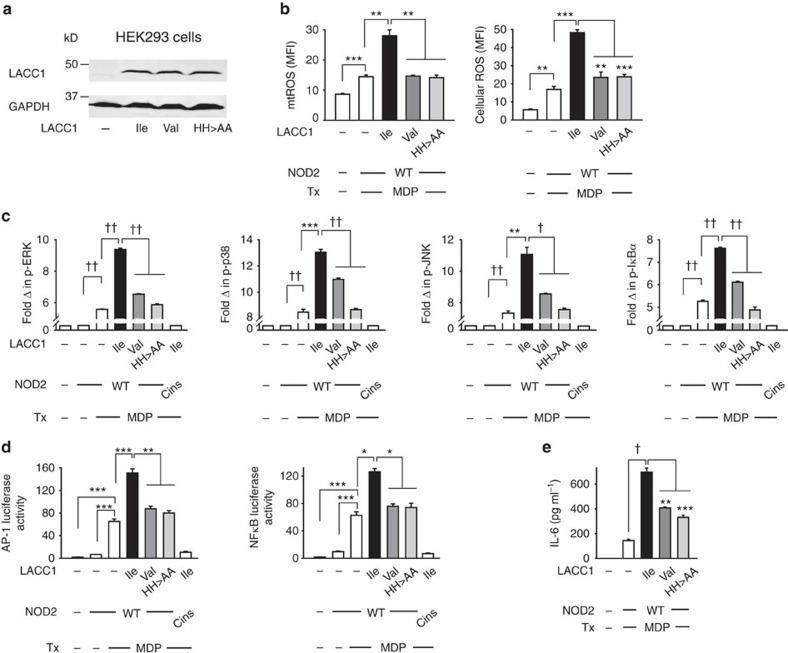
Transfection of the LACC1 risk variant results in decreased NOD2-induced outcomes. (**a**–**e**) Empty vector (EV), LACC1 Ile254 or LACC1 Val254 variants, or LACC1 His249,250Ala mutants were transfected into HEK293 cells along with NOD2±AP-1 or NFκB luciferase and Renilla constructs. (**a**) Western blot for LACC1 expression of transfected cells in one of four replicates. (**b**–**e**) Transfected cells were treated with 100 μg ml^−1^ MDP and assessed for: (**b**) mtROS and cellular ROS at 6 h, (**c**) phospho-protein induction normalized to untreated, EV-transfected cells at 15 min as assessed by flow cytometry, (**d**) AP-1 and NFκB luciferase activity at 6 h and (**e**) IL-6 secretion at 24 h. For **c**,**d** included is also the Crohn's disease-associated NOD2 LeufsinsC (Cins) variant co-transfected with LACC1 Ile254 as a control for the specificity of NOD2 responsivity to MDP treatment. Represented is mean+s.e.m. for three replicates. Panels **b**,**d**,**e** are representative of two independent experiments. Tx, treatment; WT, wild type. *P<0.05; **P<0.01; ***P<0.001; ^†^*P*<1 × 10^−4^; ^††^*P*<1 × 10^−5^; determined by 2-tailed *t*-test.

We next sought to define structural requirements in LACC1 for the LACC1-mediated mechanisms we had identified to contribute to PRR-induced outcomes. Human LACC1 is a 430-amino-acid protein, and has a sequence identity ranging from ∼85% in dogs to ∼34% in bacteria; the sequence identity of the human laccase domain (also referred to as the Cu-oxidase-4 domain) within LACC1 ranges from ∼91% to ∼33% over these same species (Supplementary Fig. 11A). Interestingly, the CD risk Val254 variant is highly conserved across species (Supplementary Fig. 11B), and the amino acid at position 254 in LACC1 is in proximity to histidines at positions 249 and 250 (Supplementary Fig. 11B,C). Crystal structures of fungal and bacterial laccase domain proteins have demonstrated a requirement for select histidines in mediating metal binding^29^,^30^,^31^. Further, the histidine at position 250 in human LACC1 is a conserved histidine (Supplementary Fig. 11B) found to form a metal-binding groove in a homologous bacterial protein through crystal structure studies^32^. To ensure that it was specifically the change from Ile to Val at the 254 amino-acid position of LACC1 that led to the modulation in PRR-induced cytokines between MDMs from LACC1 Ile254 versus LACC1 Val254 carriers (Fig. 1 and Supplementary Fig. 1), we generated both LACC1 Ile254 and LACC1 Val254 variants. Moreover, to assess the role of the nearby histidines, which we hypothesized to be required for optimal LACC1 function, we generated LACC1 in which the histidines at positions 249 and 250 were mutated to alanines. We transfected the LACC1 Ile254, Val254 and His249,250Ala variants into HEK293 cells, which did not express LACC1 (Fig. 5a), along with NOD2, so as to assess their regulation of NOD2-induced outcomes. The LACC1 variants were expressed at equal levels in HEK293 cells (Fig. 5a), indicating that neither the Ile254Val nor the His249,250Ala mutations altered LACC1 expression. LACC1 Ile254 increased NOD2-induced mtROS and cellular ROS generation (Fig. 5b), MAPK and NFκB activation (Fig. 5c), AP-1 and NFκB transcriptional activity (Fig. 5d), and IL-6 secretion (Fig. 5e). Neither the Val254 variant nor the His249,250Ala variant enhanced these NOD2-induced outcomes as effectively (Fig. 5b–e). To confirm that the signalling and transcriptional activity observed upon MDP treatment was mediated by NOD2, we used a NOD2 construct with the loss-of-function LeufsinsC mutation associated with CD. As expected, signalling and AP-1 and NFκB transcriptional activity were significantly decreased upon MDP treatment with this NOD2 variant in combination with LACC1 Ile254 (Fig. 5c,d). We further confirmed the AP-1 and NFκB transcriptional results at a lower dose of MDP treatment (Supplementary Fig. 12). Therefore, consistent with the decreased PRR-induced cytokine secretion in primary MDMs from LACC1 Val254 carriers, mutating Ile254 to Val254 in LACC1, as well as mutating the nearby histidines, led to decreased NOD2-induced mtROS, signalling and cytokine secretion in cell lines transfected with these variants.

### LACC1 mediates optimal intracellular bacterial clearance

**Figure 6:**
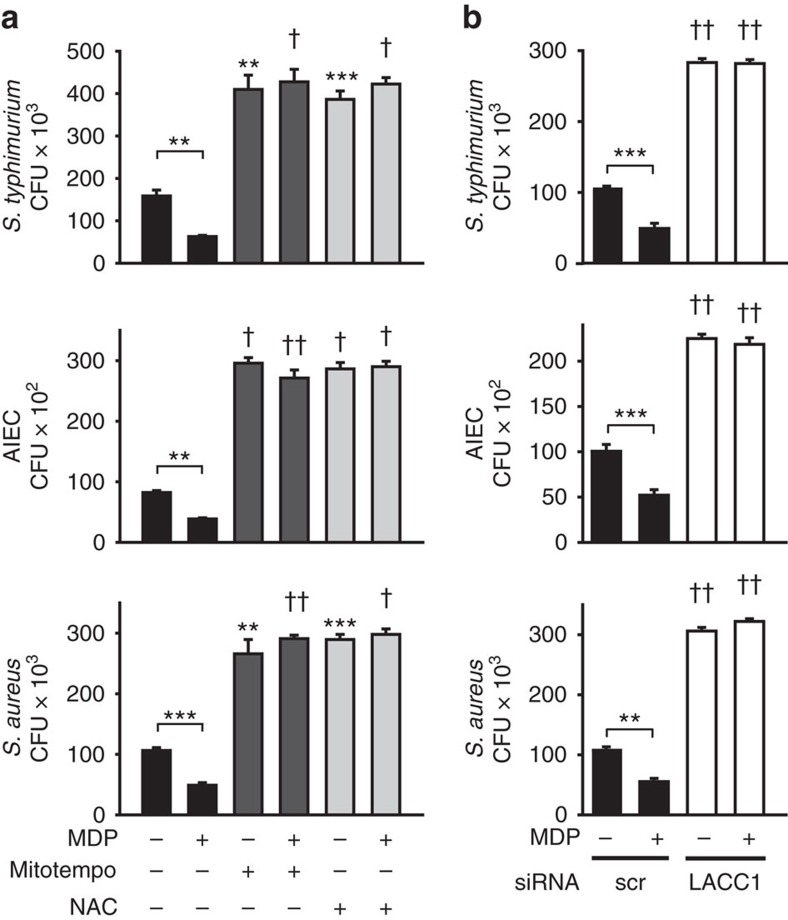
LACC1 is required for optimal NOD2-induced intracellular microbial clearance. (**a**) MDMs (*n*=4) were treated with 100 μg ml^−1^ MDP for 48 h, and then treated for 1 h with Mito-Tempo (to inhibit mtROS) or N-acetylcysteine (NAC; to neutralize ROS), and co-cultured with *S. typhimurium*, AIEC or *S. aureus*. (**b**) MDMs (*n*=4) were transfected with scrambled or LACC1 siRNA, and then treated with 100 μg ml^−1^ MDP for 48 h, followed by co-culture with *S. typhimurium*, AIEC or *S. aureus*. Similar results were observed in an additional *n*=8. Clearance of intracellular bacteria is represented as colony-forming units (CFU)+s.e.m. Significance is compared with the equivalent treatment condition in the absence of inhibitors for **a** or in scrambled siRNA-transfected cells for **b**, or as indicated. In a subset of individuals we simultaneously confirmed reduced LACC1 expression with LACC1 siRNA by western blot (Supplementary Fig. 13A) and flow cytometry. Scr, scrambled. **P<0.01; ***P<0.001; ^†^*P*<1 × 10^−4^; ^††^*P*<1 × 10^−5^; determined by 2-tailed *t*-test.

In addition to their contribution to signalling pathways, mtROS and ROS can play an important role in microbial clearance^33^. Given the critical role of microbial clearance in intestinal immune homeostasis, and that mechanisms contributing to impaired bacterial clearance can enhance risk for human IBD^3^,^4^,^5^, we questioned whether LACC1 contributes to bacterial clearance. Intestinal macrophages demonstrate enhanced microbial clearance relative to peripheral macrophages^34^. The chronic stimulation through PRRs, including through NOD2, that occurs in the intestinal environment contributes to this enhanced microbial clearance^35^. We therefore considered LACC1 contributions to microbial clearance in unstimulated MDMs and in MDMs after prolonged NOD2 stimulation, with a focus on intestinal-relevant bacteria, given the association of LACC1 with CD. We first confirmed that both mtROS and ROS are required for optimal intracellular clearance of bacteria in both unstimulated macrophages and chronic NOD2-stimulated macrophages. The mtROS and ROS requirement was observed for clearance of the intestinal pathogen *Salmonella typhimurium*, adherent invasive *Escherichia coli* (AIEC), which are enriched in the ilea of CD patients^36^, and *Staphylococcus aureus* (Fig. 6a). Knockdown of LACC1 impaired the intracellular clearance of these bacteria in unstimulated macrophages, as well as the enhanced bacterial clearance observed after the chronic NOD2 stimulation that simulates conditions in the intestinal environment (Fig. 6b). We further confirmed the role of NOD2 under these MDP-treated conditions through both NOD2 and RIP2 knockdown (Supplementary Fig. 13B). Therefore, LACC1 is required for the enhanced intracellular bacterial clearance observed in macrophages after chronic NOD2 stimulation.

### LACC1 amplifies PRR-induced succinate dehydrogenase activity

**Figure 7:**
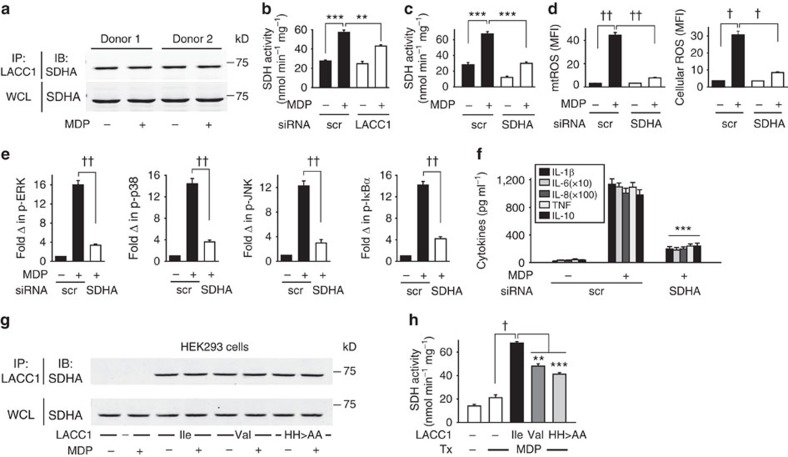
LACC1 associates with SDHA and contributes to NOD2-induced SDH activity. (**a**) MDMs were left untreated or were treated with 100 μg ml^−1^ MDP for 6 h. Cell lysates were immunoprecipitated (IP) with anti-LACC1 antibody. Associated SDHA was assessed by western blot (IB). Representative western blot in two of four individuals. (**b**) MDMs (*n*=4) were transfected with scrambled or LACC1 siRNA, and then treated with 100 μg ml^−1^ MDP and assessed for SDH activity at 2 h. Similar results were observed in an additional *n*=8. In a subset of individuals we simultaneously confirmed reduced LACC1 expression with LACC1 siRNA by western blot (Supplementary Fig. 14A) and flow cytometry. (**c**–**f**) MDMs were transfected with scrambled or SDHA siRNA, and then treated with 100 μg ml^−1^ MDP and assessed for: (**c**) SDH activity at 2 h (*n*=4), (**d**) mtROS and cellular ROS at 6 h (*n*=4; similar results in an additional *n*=4), (**e**) fold phospho-protein induction normalized to untreated cells at 15 min (*n*=4) as assessed by flow cytometry, (**f**) cytokines at 24 h (*n*=4). (**g**,**h**) LACC1 Ile254, Val254 and His249,250Ala variants were transfected into HEK293 cells along with NOD2, and cells were then treated with 100 μg ml^−1^ MDP. (**g**) Cell lysates were immunoprecipitated (IP) with anti-LACC1 antibody at 6 h. Associated SDHA was assessed by western blot (IB). Shown is a representative western blot from one of three replicates. (**h**) SDH activity was assessed. Data represent three replicates and were repeated two independent times. Shown is mean+s.e.m. for (**b**–**f**,**h**). WCL, whole cell lysate; Tx, treatment; scr, scrambled. **P<0.01; ***P<0.001; ^†^*P*<1 × 10^−4^; ^††^*P*<1 × 10^−5^; determined by 2-tailed *t*-test.

We next sought to further define the mechanisms through which LACC1 modulates PRR-induced mtROS, and in turn, subsequent signalling and cytokine secretion. We considered mitochondrial enzymes critical in the generation of mtROS with which LACC1 might interact. SDHA is localized in the inner mitochondrial membrane and is a major subunit of succinate-ubiquinone oxidoreductase, a complex of the mitochondrial respiratory chain important for mtROS generation^37^,^38^,^39^, such that we considered SDHA as a candidate through which LACC1 might cooperate with to modulate mtROS. Endogenous LACC1 and SDHA associated with each other in human MDMs (Fig. 7a); this association was not modulated by NOD2 stimulation (Fig. 7a). Importantly, succinate dehydrogenase (SDH) activity increased with NOD2 stimulation and this increase was impaired upon LACC1 knockdown (Fig. 7b). Through SDHA knockdown (Supplementary Fig. 14B,C), we confirmed that SDHA was required for optimal NOD2-induced SDH activity (Fig. 7c), mtROS and cellular ROS (Fig. 7d), signalling (Fig. 7e) and cytokine secretion (Fig. 7f). Cells were viable with SDHA knockdown (Supplementary Fig. 14D). Having identified a role for LACC1 in increasing SDH activity in human MDMs, we next questioned whether the generated LACC1 mutants regulated these effects. In HEK293 cells transfected with LACC1 Ile254, LACC1 associated with SDHA constitutively (Fig. 7g). Similar to primary MDMs (Fig. 7a), this association was not altered with NOD2 stimulation (Fig. 7g). However, SDH enzyme activity increased with NOD2 stimulation in the presence of LACC1 Ile254 (Fig. 7h). The LACC1 Val254 and LACC1 His249,250Ala variants did not lead to altered association with SDHA relative to the LACC1 Ile254 variant (Fig. 7g). However, in comparison to the LACC1 Ile254 variant, the LACC1 Val254 disease-risk variant and the LACC1 His249,250Ala mutant demonstrated less effective NOD2-induced SDH activity upon transfection into HEK293 cells (Fig. 7h). Therefore, LACC1 associates with SDHA and cooperates to enhance NOD2-induced SDH activity, with the SDHA pathway leading to increased mtROS and cytokine secretion.

### LACC1 contributes to NOD2-induced complex assembly

As the LACC1 association with SDHA is constitutive rather than inducible (Fig. 7), we sought to identify NOD2-induced post-translational modifications (for example, phosphorylation, acetylation) on LACC1 that might be contributing to LACC1 function, or additional upstream protein associations or outcomes that might be mediating its regulation of mtROS, ROS, signalling and cytokine secretion with NOD2 stimulation. Upon NOD2 stimulation we observed tyrosine phosphorylation of a band corresponding to the molecular weight of LACC1 and of proteins associated in a complex with LACC1 (Supplementary Fig. 15A). We identified three tyrosine motifs at positions 52, 89 and 265 within LACC1 that demonstrated a high probability (>0.5 by NetPhos 3.1) of serving as phosphorylation sites. We generated LACC1 constructs in which each of these tyrosine sites was mutated alone or in combination. However, upon transfection into HEK293 cells, none of these tyrosine LACC1 mutants demonstrated less NOD2-induced cytokine secretion relative to the LACC1 Ile254 variant (Supplementary Fig. 15B). We next identified a lysine at position 247 with predicted acetylation. We generated a LACC1 K247A construct and transfected the LACC1 K247 and A247 constructs into HEK293 cells. Upon stimulation of NOD2 in these HEK293-transfected cells, acetylation was observed at a band corresponding to the molecular weight of LACC1 in the LACC1 K247 variant and this acetylation was attenuated with the LACC1 A247 mutation (Supplementary Fig. 15C). However, NOD2-induced cytokines were not altered despite the loss of this post-translational modification (Supplementary Fig. 15D). Therefore, neither phosphorylation of select tyrosine residues nor lysine acetylation in LACC1 was required for NOD2-induced cytokines.

**Figure 8:**
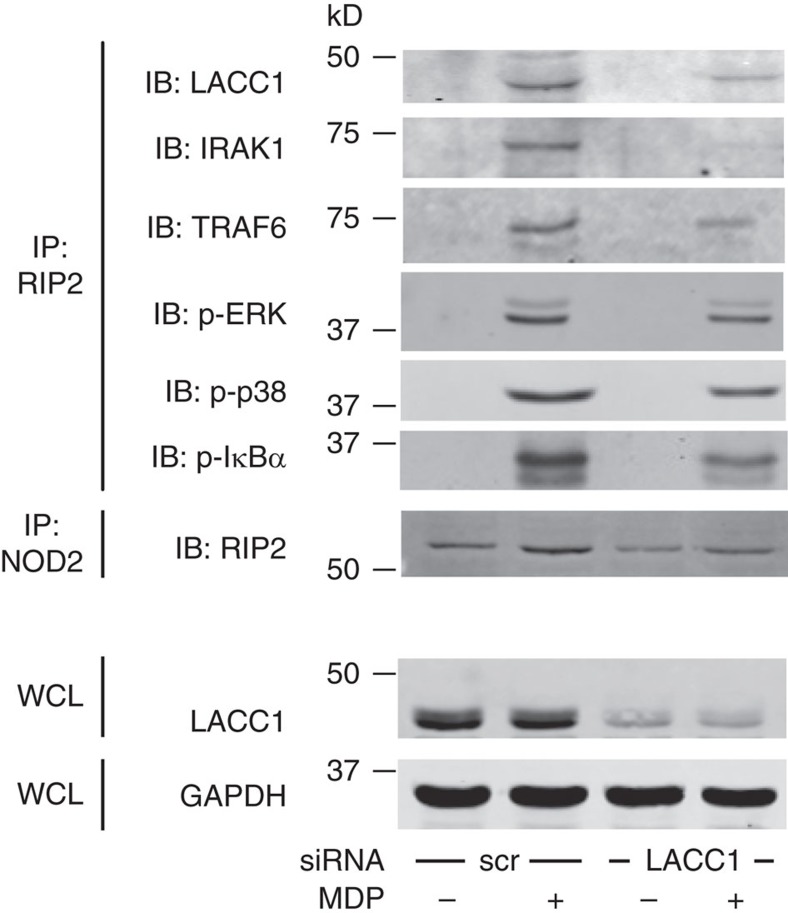
LACC1 is recruited to a NOD2-induced signalling complex. MDMs were transfected with scrambled or LACC1 siRNA, and then treated with 100 μg ml^−1^ MDP for 15 min. RIP2 or NOD2 were immunoprecipitated (IP) from cell lysates and the recruitment of the indicated proteins was assessed by western blot (IB). GAPDH was used as a loading control. WCL, whole-cell lysate; scr, scrambled.

We next considered that LACC1 might be associating in a complex with additional key cytoplasmic proteins in a NOD2-inducible manner, which are, in turn, required for NOD2-induced mtROS, signalling and cytokines. NOD2 can associate with RIP2 and signal through IRAK1 and TRAF6 pathways upon MDP treatment^40^,^41^,^42^. Upon NOD2 stimulation, LACC1 was recruited to a complex consisting of NOD2, RIP2, IRAK1 and TRAF6, as well as phospho-ERK, phospho-p38 and phospho-IκBα (Fig. 8). We observed similar association with the proximal complex intermediates using a reciprocal immunoprecipitation approach (Supplementary Fig. 16A). With LACC1 knockdown, the assembly of this complex was impaired (Fig. 8). Each intermediate in this complex was required for optimal NOD2-induced mtROS, ROS, signalling, cytokines and bacterial clearance (Supplementary Figs 10C–E, 13B and 16B–E). Therefore, LACC1 associates with and is required for the optimal assembly of NOD2-signalling intermediates following NOD2 stimulation.

### Polyphenol oxidase activity contributes to PRR outcomes

We sought to further define whether a subset of LACC1-regulated functions observed in human MDMs might be attributable to the enzymatic activity exhibited by this family of proteins. Bacterial and fungal laccase-containing proteins can oxidize polyphenols^13^. Mitochondrial-targeted polyphenols can, in turn, modulate mtROS levels^38^,^39^. We were unable to observe polyphenol oxidase activity *in vitro* with human LACC1 protein purified under a variety of conditions and assessed against multiple different laccase substrates. In contrast, we observed copper-dependent polyphenol oxidase activity in side-by-side studies utilizing *E. coli* CueO laccase, which we generated as a positive control, as well as activity utilizing a fungal laccase from *Rhus vernicifera* as has been previously described^30^,^43^.

**Figure 9:**
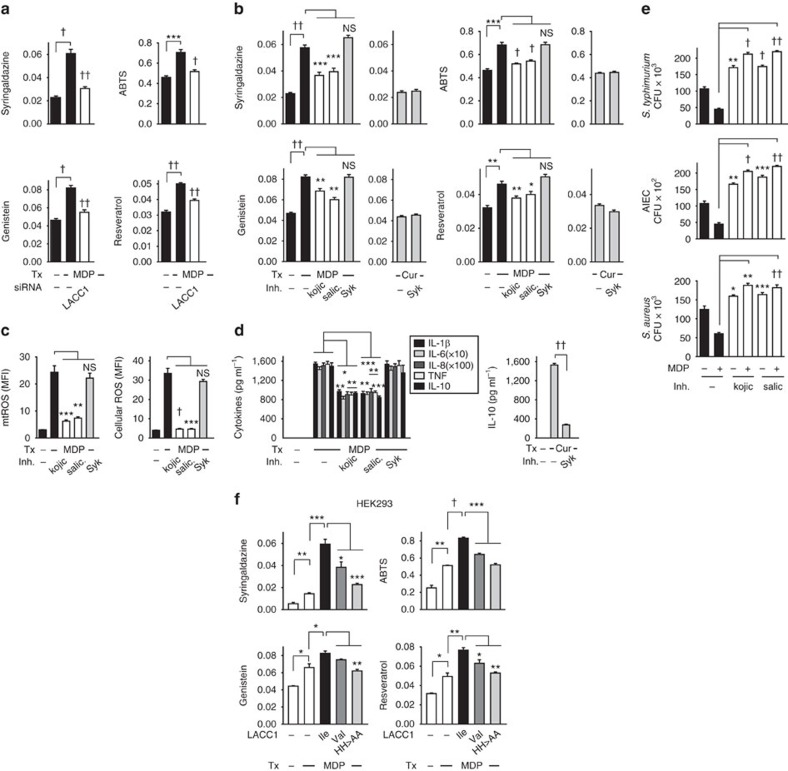
LACC1 regulates NOD2-induced polyphenol oxidase activity in MDMs. (**a**) MDMs were transfected with scrambled or LACC1 siRNA, and then treated with 100 μg ml^−1^ MDP for 6 h and polyphenol oxidase activity was assessed in cell lysates utilizing syringaldazine, ABTS, genistein and resveratrol as substrates (*n*=4). Similar results were seen in an additional *n*=4 for syringaldazine and ABTS. (**b**–**e**) MDMs were preincubated with kojic acid, salicylhydroxamic acid (polyphenol oxidase inhibitors) or a Syk inhibitor for 1 h, and then stimulated with 100 μg ml^−1^ MDP and assessed for: (**b**) polyphenol oxidase activity at 6 h (*n*=8, similar results were seen in an additional *n*=4), (**c**) mtROS and cellular ROS at 6 h (*n*=6, similar results were seen in an additional *n*=4), (**d**) cytokines at 24 h (*n*=4, similar results were seen in an additional *n*=8) and (**e**) bacterial clearance (*n*=4; significance is compared to non-MDP-treated MDMs without inhibitor treatment or as indicated). Curdlan (cur) treatment is used as a control in **b**,**d**. (**f**) LACC1 Ile254, Val254 or His249,250Ala variants were transfected into HEK293 cells along with NOD2. Cells were then treated with 100 μg ml^−1^ MDP for 6 h. Polyphenol oxidase activity was assessed. Data represent three replicates repeated two independent times. Inh, inhibitors; salic, salicylhydroxamic acid; Tx, treatment. *P<0.05; **P<0.01; ***P<0.001; ^†^*P*<1 × 10^−4^; ^††^*P*<1 × 10^−5^; NS, not significant; determined by 2-tailed *t*-test.

We next postulated that human polyphenol oxidase activity would likely be tightly regulated within the cellular environment in which it is functioning, such that we sought to detect this activity under appropriate stimulation conditions in cells. We therefore examined if polyphenol oxidation was regulated by NOD2 stimulation in human MDMs, and if this, in turn, was modulated by LACC1. We observed increased oxidation of two common laccase substrates, syringaldazine and 2,2′-azino-bis(3-ethylbenzothiazoline-6-sulfonic acid) (ABTS), by cellular lysates from NOD2-stimulated MDMs (Fig. 9a). We further observed oxidation of two mitochondrial-associated polyphenols^44^,^45^ with NOD2 stimulation, genistein and resveratrol (Fig. 9a). Furthermore, this NOD2-induced polyphenol oxidase activity was impaired with LACC1 knockdown (Fig. 9a). To determine whether polyphenol oxidase activity contributed to the LACC1-dependent outcomes we observed in MDMs, we identified two polyphenol oxidase inhibitors, kojic acid and salicylhydroxamic acid, used in prior studies to inhibit laccase-containing proteins^46^,^47^. We ensured that these inhibitors directly inhibited polyphenol oxidase activity *in vitro* (Supplementary Fig. 17A). As a negative control, we utilized a Syk inhibitor (Supplementary Fig. 17A), since Syk is activated downstream of dectin and we had identified that dectin functioned in a LACC1-independent manner (Supplementary Figs 2C and 6C,D). Cells were viable when treated with the polyphenol oxidase inhibitors and remained responsive to stimulation through dectin (Supplementary Fig. 17B,C). We found that both these inhibitors decreased NOD2-induced polyphenol oxidase activity in cells as assessed by the decreased ability to oxidize polyphenol substrates (Fig. 9b). In contrast, dectin stimulation did not induce polyphenol oxidase activity (Fig. 9b). We further found that with inhibition of polyphenol oxidase activity in MDMs, NOD2-induced mtROS, ROS, cytokines and bacterial clearance was significantly decreased (Fig. 9c–e). We next questioned whether the generated LACC1 mutants regulated NOD2-induced cellular polyphenol oxidase activity. HEK293 cells transfected with LACC1 Ile254 demonstrated a significant increase in NOD2-induced polyphenol oxidase activity, whereas the LACC1 Val254 disease-risk variant and the LACC1 His249,250Ala mutant demonstrated less effective NOD2-induced polyphenol oxidase activity (Fig. 9f).

### LACC1 Val254 MDMs have lower NOD2-induced outcomes

**Figure 10:**
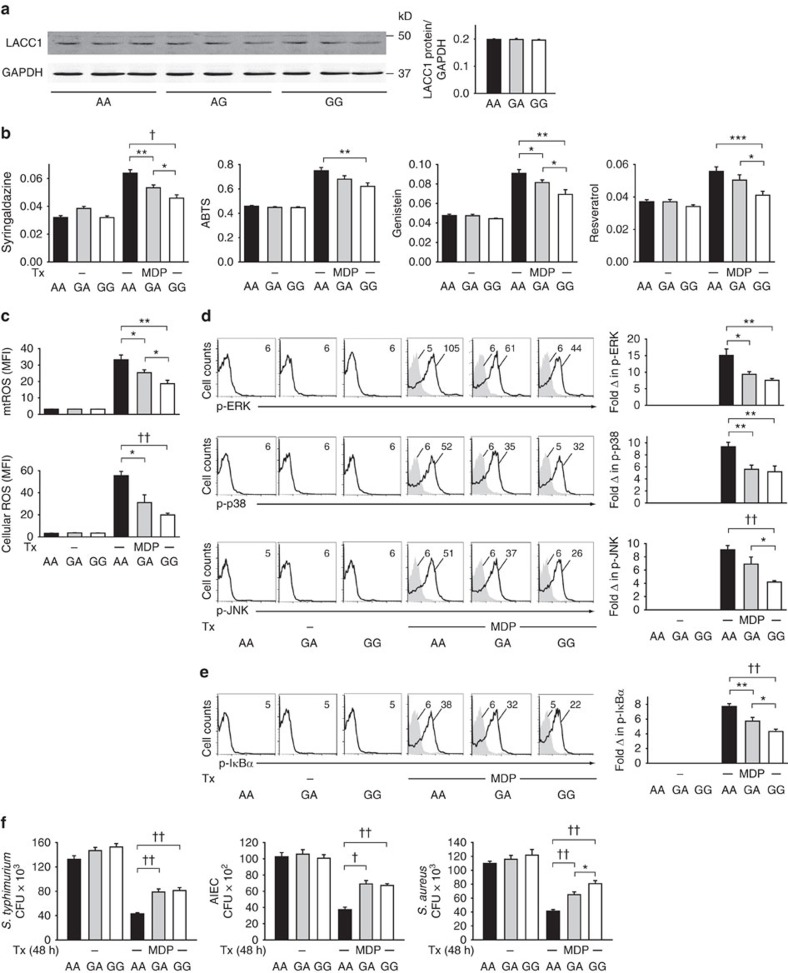
LACC1 Val254 risk carrier MDMs show reduced LACC1-dependent outcomes with NOD2 stimulation. MDMs from LACC1 Ile254, Ile/Val254 and Val254 carriers (rs3764147 AA, GA and GG carriers, respectively) were treated with 100 μg ml^−1^ MDP and assessed for: (**a**) LACC1 expression at 12 h. Representative western blot and summary of densitometry+s.e.m. for *n*=9 per genotype. (**b**) Polyphenol oxidase activity at 6 h (*n*=15 per genotype). Mean+s.e.m. (**c**) mtROS (*n*=8 per genotype) and cellular ROS (*n*=7 per genotype) at 6 h. Mean+s.e.m. (**d**) Phospho-ERK, phospho-p38 and phospho-JNK, and (**e**) phospho-IκBα at 15 min with representative flow cytometry plots showing MFI values and summarized data represented as fold phospho-protein induction normalized to untreated cells+s.e.m. (*n*=8 per genotype). (**f**) Intracellular clearance of *S. typhimurium*, AIEC or *S. aureus* at 48 h. CFU+s.e.m. (*n*=10 per genotype). Tx, treatment. *P<0.05; **P<0.01; ***P<0.001; ^†^*P*<1 × 10^−4^; ^††^*P*<1 × 10^−5^; determined by 2-tailed *t*-test.

We next questioned whether the rs3764147 polymorphism in *LACC1* (resulting in an Ile254Val mutation in G risk carriers) led to genotype-dependent regulation of the LACC1 functions we had identified to modulate PRR-initiated outcomes. We first assessed whether MDMs from LACC1 Ile254, Val254 or heterozygote carriers demonstrated differences in LACC1 expression. LACC1 protein expression was equivalent between MDMs from these individuals (Fig. 10a), consistent with our findings that transfection of LACC1 Ile254 and Val254 variants into HEK293 cells expressed at equivalent levels (Fig. 5a). The recruitment of LACC1 to and assembly of the complex of proximal intermediates upon NOD2 stimulation was also equivalent between LACC1 Ile254 and Val254 carrier MDMs (Supplementary Fig. 18). In contrast, and consistent with the decreased NOD2-induced polyphenol oxidase activity, mtROS and ROS production, signalling and cytokines in LACC1 Val254-transfected HEK293 cells (Figs 5 and 9f), MDMs expressing the endogenous LACC1 Val254 variant demonstrated lower NOD2-induced polyphenol oxidase activity (Fig. 10b), mtROS and cellular ROS production (Fig. 10c), and activation of the MAPK (Fig. 10d) and NFκB (Fig. 10e) pathways relative to LACC1 Ile254 MDMs. These results are consistent with the decreased NOD2- and PRR-initiated cytokine secretion observed in MDMs from LACC1 Val254 carriers (Fig. 1 and Supplementary Fig. 1). Heterozygote individuals were intermediate or similar to homozygote Val254 risk individuals in the measures assessed (Fig. 10b–e), also consistent with the PRR-initiated cytokine secretion results in Fig. 1. Finally, consistent with the lower NOD2-induced mtROS and ROS production in MDMs from LACC1 Val254 carriers, MDMs from LACC1 Val254 carriers demonstrated decreased intracellular clearance of *S. typhimurium*, AIEC and *S. aureus* after chronic NOD2 stimulation (Fig. 10f). Therefore, relative to LACC1 Ile254 carriers, MDMs from LACC1 Val254 risk carriers demonstrate decreased NOD2-induced polyphenol oxidase activity, mtROS and cellular ROS, downstream signalling, cytokine secretion and intracellular bacterial clearance.

## Discussion

We establish that LACC1 is expressed in human peripheral and intestinal myeloid-derived cells, define roles for human LACC1 in amplifying PRR-induced outcomes in macrophages, dissect mechanisms wherein LACC1 contributes to PRR outcomes and determine that the CD- and leprosy-associated LACC1 Val254 risk variant results in lower PRR-initiated polyphenol oxidase activity, mtROS and cellular ROS, signalling, cytokine secretion, broad transcriptional regulation and intracellular bacterial clearance (Supplementary Fig. 19). We established a number of mechanisms through which LACC1 regulates these outcomes, including its contribution to the optimal assembly of a complex of signalling intermediates induced upon NOD2 stimulation, and its ability to regulate cellular polyphenol oxidase activity and SDH enzymatic activity. There are likely other mitochondrial-dependent and -independent mechanisms through which LACC1 might modulate both PRR-dependent and -independent functions in macrophages, as well as other cell subsets in which LACC1 might regulate distinct outcomes. Increased inflammation associated with loss-of-function in innate pathway outcomes is commonly observed; decreased innate function can result in an inefficient elimination of microbes, thereby leading to compensatory inflammatory responses^48^,^49^,^50^. As such, we observe that LACC1 is required for the enhanced microbial clearance observed after chronic NOD2 stimulation, conditions that simulate those in the intestinal environment; intestinal macrophages from NOD2-deficient mice demonstrate impaired microbial clearance^35^. In addition to the attenuated production of mtROS and cellular ROS that we now identify in *LACC1* risk carriers, decreased ROS production has been observed with NADPH oxidase complex variants associated with early-onset IBD^4^, and with chronic granulomatous disease, a disease in which a substantial percentage of individuals develop intestinal inflammation^5^. In contrast to the decreased responses observed with CD-associated *NOD2*, the LACC1 Val254 risk variant decreases responses through a broad range of PRRs. Our findings identify a role for human LACC1, and implicate the *LACC1* disease-risk variant as a loss-of-function in PRR-initiated mtROS, signalling, cytokines and bacterial clearance, thereby highlighting pathways that might ultimately be modulated therapeutically in disease.

## Methods

### Patient recruitment and genotyping

Informed consent was obtained as per the protocol approved by the institutional review board at Yale University, and healthy controls were recruited for peripheral blood draws. Given the limitation in peripheral cell numbers and the range of innate responses we sought to examine, two separate cohorts of 100 and 98 individuals were recruited for NOD2/TLR2 dose–response studies in MDMs, and PRR-induced cytokine studies in MDDCs, respectively. We performed genotyping by TaqMan SNP genotyping (Applied Biosystems, Foster City, CA) or Sequenom platform (Sequenom Inc., San Diego, CA).

### Myeloid cell isolation and cell culture

Monocytes were purified from human peripheral blood mononuclear cells by positive CD14 selection (Miltenyi Biotec, Auburn, CA) or by adhesion, tested for purity and cultured with 10 ng ml^−1^ macrophage colony-stimulating factor (M-CSF; Shenandoah Biotechnology, Warwick, PA; for MDM differentiation) or 40 ng ml^−1^ GM-CSF and 40 ng ml^−1^ IL-4 (R&D Systems Inc., Minneapolis, MN; for MDDC differentiation) as in ref. 42. Cultured myeloid cells were treated with MDP (Bachem, King of Prussia, PA), Pam3Cys (EMD Millipore, Billerica, MA), lipid A (Peptides International, Louisville, KY), TriDAP or flagellin (Invivogen, San Diego, CA). In some experiments, MDMs were treated with polyphenol oxidase inhibitors (5 μM kojic acid, 1 mM salicylhydroxamic acid (VWR, Radnor, PA)) or a Syk inhibitor (1 μM; ThermoFisher Scientific, Waltham, MA). Supernatants were assayed for TNF (clones MAb1 and MAb11, diluted 1:1,000), IL-6 (clones MQ2-13A5 and MQ2-39C3, diluted 1:1,000), IL-8 (clones G265-5 and G265-8, diluted 1:1,000), IL-10 (clones JES3-9D7 and JES3-12G8, diluted 1:1,000; BD Biosciences) or IL-1β (clones CRM56 and CRM57 diluted 1:1,000; eBioscience, San Diego, CA) by ELISA. Myeloid cells (CD11c purity >75%) were isolated as in ref. 51 from colonic resection specimens from uninvolved intestine in seven non-IBD patients undergoing surgery for diverticular disease or colon cancer.

### mRNA expression analysis

RNA was isolated, reverse-transcribed and quantitative PCR performed on the ABI Prism 7000 (Applied Biosystems). Samples were normalized to GAPDH. Primer sequences are included in Supplementary Table 1.

### Transfection of siRNAs and DNA vectors

Pooled siRNA containing four different siRNAs for each LACC1 and SDHA (SMARTpool, Dharmacon, Lafayette, CO), or scrambled siRNA (Dharmacon) were transfected into MDMs using a nucleofector kit (Amaxa, San Diego, CA) for 48 h unless otherwise indicated. The LACC1 Ile254 variant was subcloned from genomic DNA into pcDNA.3. pcDNA.3-LACC1 (Val254 variant) and pcDNA.3-LACC1 (His249,250Ala) were both generated through site-directed mutagenesis (QuikChange Lightning Kit; Agilent Technologies) of the LACC1 parent vector and confirmed by sequencing. HEK293 cells were transiently transfected by Lipofectamine (Invitrogen) with 1 μg of each of the LACC1 constructs or empty vector ±50 ng wild-type NOD2 (pcDNA.3) or NOD2 LeufsinsC ±50 ng pAP-1-luciferase or pNFκB luciferase (Clontech, Mountain View, CA) along with 15 ng pRL-TK (Promega, Madison, WI) as a Renilla normalization control. Twenty-four hours after transfection, cells were treated with MDP and assessed for the indicated measures.

### Luciferase activity

Cells were lysed in 1 × passive lysis buffer, scraped, pelleted and then assayed for luciferase and Renilla activity (Promega) according to the manufacturer's instructions and using the Synergy 2 plate reader (Bioteck, Winooski, VT).

### Protein expression analysis

Western blot was performed using anti-LACC1 antibodies (Abcam, diluted 1:1,000). Loading controls included GAPDH (EMD Millipore, clone 6C5, diluted 1:10,000) for cell lysates, α-tubulin (Cell Signaling, diluted 1:1,000) for cytoplasmic protein and MTCO2 (Abcam, clone MTC02, diluted 1:1,000) for mitochondrial proteins. Mitochondrial and cytoplasmic subcellular fractionation was conducted using the Mitochondria isolation kit (ab 110170, Abcam). LACC1 (with anti-LACC1, Santa Cruz Biotechnology, using 4 μg antibody), RIP2 (with anti-RIP2, BD Biosciences, clone 25/RIG-G, using 4 μg antibody) or NOD2 (with anti-NOD2, Cayman Chemical, using 4 μg antibody) were immunoprecipitated from transfected HEK293 cells or MDMs with antibody-bound protein A sepharose beads. Associated proteins were examined with anti-SDHA (Abcam, clone 2E3GC12FB2A[E2], diluted 1:500), anti-LACC1 (Santa Cruz Biotechnology, diluted 1:1,000), anti-IRAK1 (clone D51G7, diluted 1:1,000), anti-TRAF6 (clone D21G3, diluted 1:1,000), anti-phospho-ERK (diluted 1:1,000), anti-phospho-p38 (clone 28B10, diluted 1:1,000) or anti-phospho-IκBα (clone 5A5, diluted 1:1,000; all Cell Signaling Technology) antibodies.

### ROS production

MtROS and ROS production was assessed by flow cytometry after incubation with 5 μM MitoSOX and 10 μM H2DCFDA (2′,7′-dichlorodihydrofluorescein diacetate; Life Technologies Corporation, Grand Island, NY) × 30 min in PBS, respectively.

### Phospho-flow

Phospho-protein induction in permeabilized cells was determined using Alexa Fluor 647-, phycoerythrin-, Alexa Fluor 488- or biotin-labelled antibodies to phospho-ERK (clone D13.14.4E, diluted 1:1,000), phospho-p38 (clone 3D7, diluted 1:1,000), phospho-JNK (clone G9, diluted 1:1,000) or phospho-IκBα (clone 14D4, diluted 1:1,000; all Cell Signaling) by flow cytometry.

### Polyphenol oxidase activity

ABTS and syringaldazine oxidation in cell lysates suspended in PBS was measured at 420 and 530 nm, respectively, as previously described^52^. Oxidation of genistein and resveratrol (ThermoFisher Scientific) was monitored at 400 and 320 nm, respectively.

### SDH activity

SDH activity was assayed in purified mitochondria resuspended in 35 mM potassium phosphate monobasic buffer (pH 7.3; ref. 53). Disodium succinate (10 mM; Sigma) and 40 μM mM 2,6-dichlorophenolindophenol sodium salt hydrate (DCPIP; Sigma) was added to the mitochondria at 37 ^o^C, and DCPIP extinction was measured at 610 nM as described in ref. 53.

### Intracellular bacterial killing.

Cells were infected with *S. typhimurium* (10:1), AIEC strain LF82 (10:1; generous gift from Dr Emiko Mizoguchi) or *S. aureus* (1:1) for a total of 2 h, with gentamicin added 1 h after bacterial infection^35^. In some cases, cells were pretreated for 1 h with 50 nM Mito-Tempo (Santa Cruz) or 20 nM N-acetylcysteine (Sigma).

### Statistical analysis

Significance was assessed using two-tailed Student's *t*-test. *P*<0.05 was considered significant. Error bars are shown as s.e.m.

### Data availability

The data supporting the findings of this study are available within the article and its Supplementary Information files.

## Additional information

**How to cite this article:** Lahiri, A. *et al*. Human LACC1 increases innate receptor-induced responses and a *LACC1* disease-risk variant modulates these outcomes. *Nat. Commun.* **8**, 15614 doi: 10.1038/ncomms15614 (2017).

**Publisher's note:** Springer Nature remains neutral with regard to jurisdictional claims in published maps and institutional affiliations.

## Supplementary Material

Supplementary Information Supplementary Figures and Supplementary Table
