# Supplementary material for: Human LACC1 increases innate receptor-induced responses and a LACC1 disease-risk variant modulates these outcomes
Source: Nat Commun. 2017 Jun 8;8:15614. doi: 10.1038/ncomms15614 (PMC5472760; doi:10.1038/ncomms15614)
Supplement: Supplementary Information — Supplementary Figures and Supplementary Table [file ncomms15614-s1.pdf]

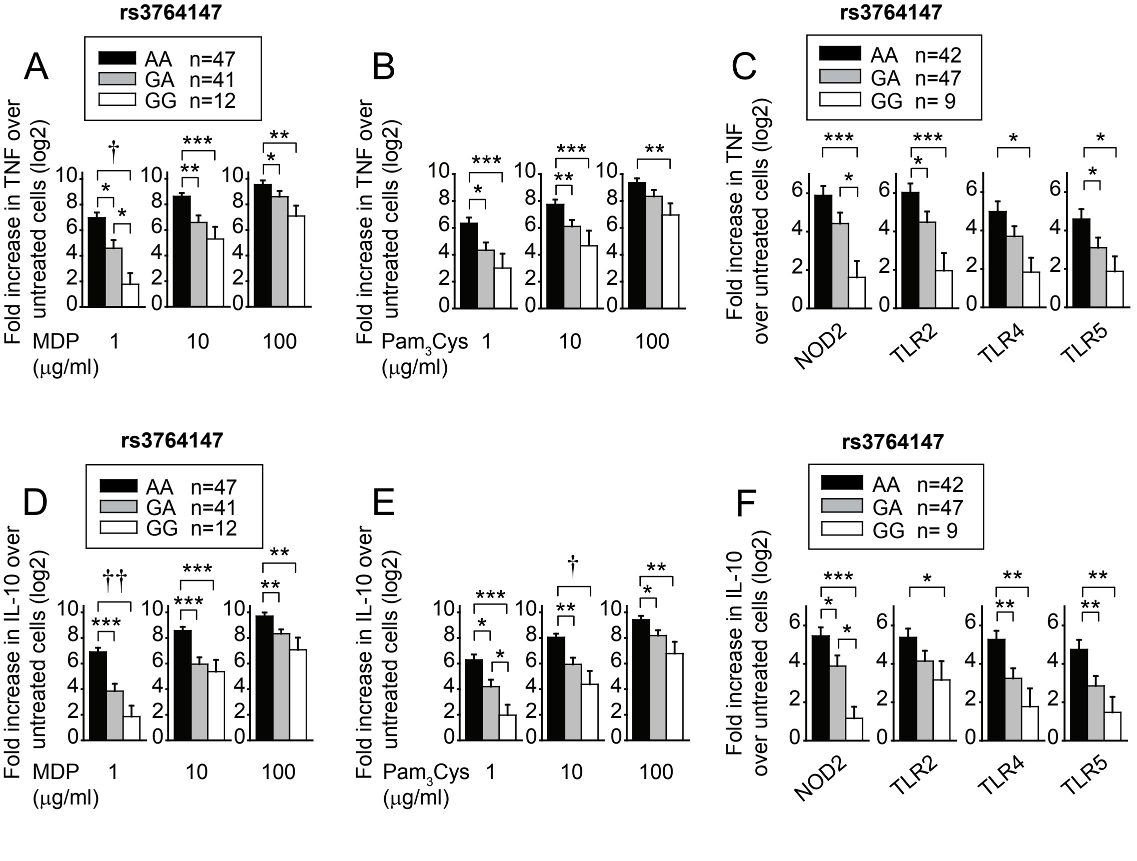

Supplementary Figure 1. Primary human myeloid cells from disease-associated rs3764147 G (Val254) risk carriers demonstrate decreased cytokine secretion upon PRR stimulation. Human MDMs (n=100) were treated for 24h with **(A, D)** 1, 10 or 100µg/ml MDP, or **(B, E)** 1, 10 or 100µg/ml Pam3Cys. **(C, F)** Human MDDCs (n=98) were treated for 24h with 1µg/ml MDP (NOD2 ligand), 1µg/ml Pam3Cys (TLR2 ligand), 0.01µg/ml lipid A (TLR4 ligand) or 0.5ng/ml flagellin (TLR5 ligand) for 24h. Shown is fold **(A-C)** TNF and **(D-F)** IL-10 secreted protein induction (log2 transformed) stratified on rs3764147 genotype + SEM. *, $p<0.05$; **, $p<0.01$; ***, $p<0.001$; †, $p<1\times10^{-4}$; ††, $p<1\times10^{-5}$.

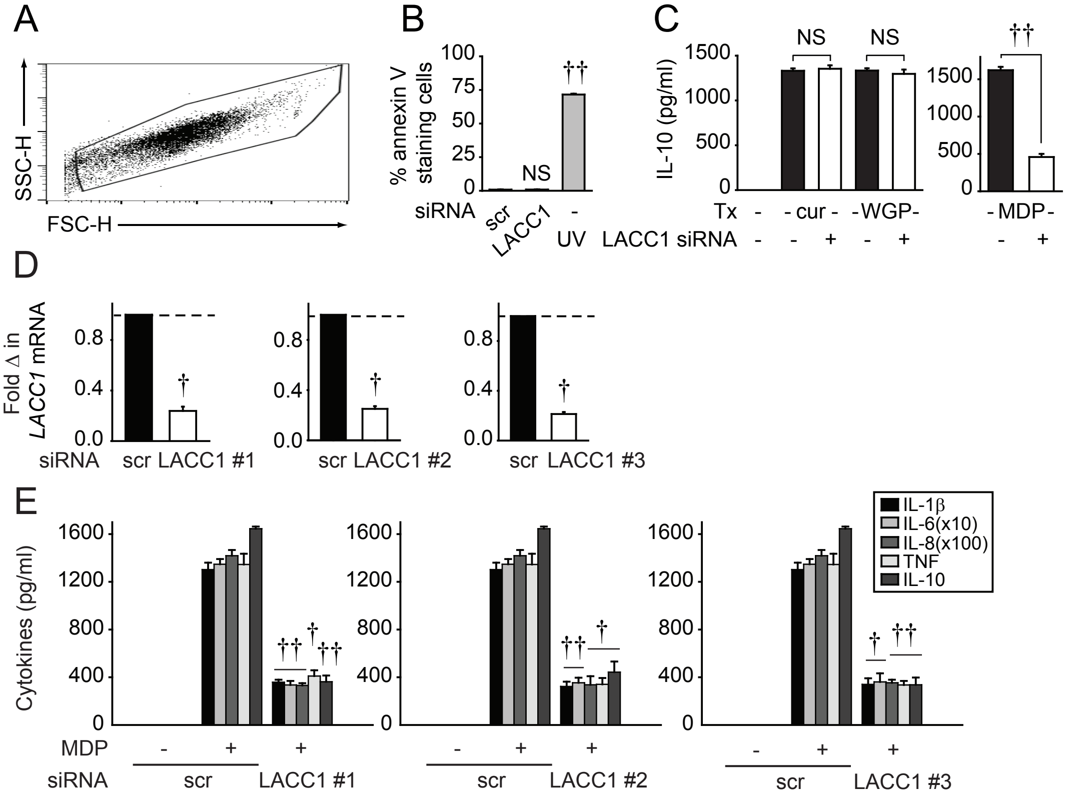

Supplementary Figure 2. MDMs remain functional after LACC1 knockdown. (A) Shown is the gating strategy for Figure 2C. **(B)** MDMs (n=4) were transfected with scrambled or LACC1 siRNA, then stained with annexin V (eBiosciences). Shown is percent annexin V^{+} cells + SEM as a measure of cell death. UV stimulation at 50-100 J/m^{2} is shown as a positive control. **(C)** MDMs (n=4) were transfected with scrambled or LACC1 siRNA, then treated with 100 µg/ml curdlan (cur) or 100 µg/ml dispersible whole glucan particles (WGP) (Invivogen, San Diego, CA) for 24h. Mean IL-10 secretion + SEM. Treatment with 100 µg/ml MDP is shown as a positive control for LACC1 knockdown effects. **(D-E)** MDMs (n=4) were transfected with scrambled or LACC1 siRNA (three different individual siRNAs: siGenome J-015653-17 [#1], J-015653-18 [#2], J-015653-19 [#3]) (Dharmacon). **(D)** *LACC1* mRNA expression normalized to scrambled siRNA-transfected cells + SEM. **(E)** Transfected MDMs were treated with 100µg/ml MDP for 24h. Cytokine secretion + SEM. Significance was calculated compared to scrambled siRNA-transfected, MDP-treated cells. Similar results were observed in an additional n=4. Scr, scrambled; NS, not significant; tx, treatment. †, $p<1x10^{-4}$; ††, $p<1x10^{-5}$.

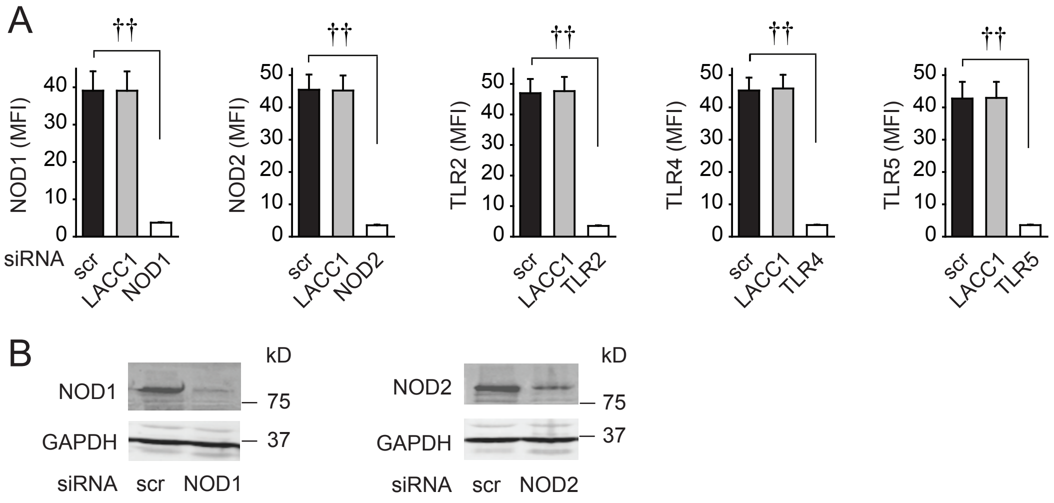

Supplementary Figure 3. LACC1 knockdown does not affect PRR expression. (A) MDMs (n=8) were transfected with scrambled or LACC1 siRNA. Protein expression of NOD1 (Novus, Littleton, CO), NOD2, TLR2 (BD Biosciences, clone 11G7, diluted 1:1000), TLR4 (eBiosciences, clone HTA125, diluted 1:1000), and TLR5 (BD Biosciences, clone 624915, diluted 1:1000) was assessed by flow cytometry and is shown as MFI + SEM. As a control for the ability to detect reduced expression of each PRR examined, siRNA to the respective PRR was also included. **(B)** MDMs were transfected with scrambled, NOD1 or NOD2 siRNA. Shown is a representative Western blot to demonstrate a second method of detecting reduced expression of the intracellular proteins NOD1 and NOD2. Scr, scrambled; ††, $p<1x10^{-5}$.

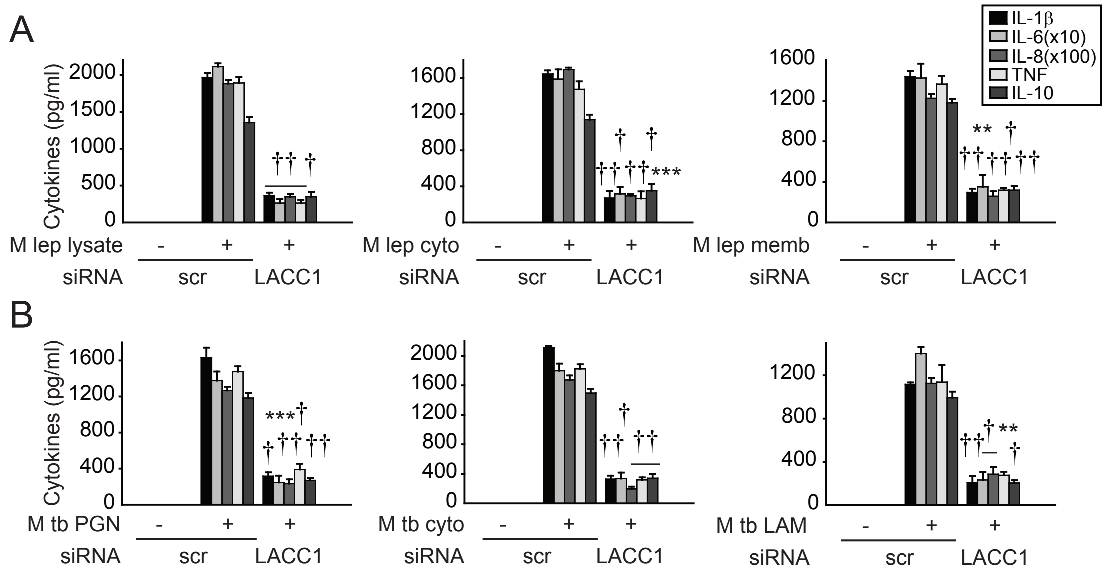

Supplementary Figure 4. LACC1 is required for optimal mycobacterial product-mediated cytokine secretion. MDMs (n=4) were transfected with scrambled or LACC1 siRNA, then treated with **(A)** 100µg/ml *M. leprae* whole cell lysate (M. lep lysate), *M. leprae* cytosolic fraction (M. lep cyto), *M. leprae* membrane fraction (M. lep memb), or **(B)** *M. tuberculosis* peptidoglycan (M. tb PGN), *M. tuberculosis* cytosolic fraction (M. tb cyto) or *M. tuberculosis* lipoarabinomannan (LAM) protein (M. tb LAM) (BEI resources, NIH Biodefense and Emerging Infections Research Resources Repository, NIAID, NIH) for 24h. Supernatants were assessed for cytokines+SEM. Similar results were observed for an additional n=8. Scr, scrambled. **, p<0.01; ***, p<0.001; †, p<1x10^{-4}; ††, p<1x10^{-5}.

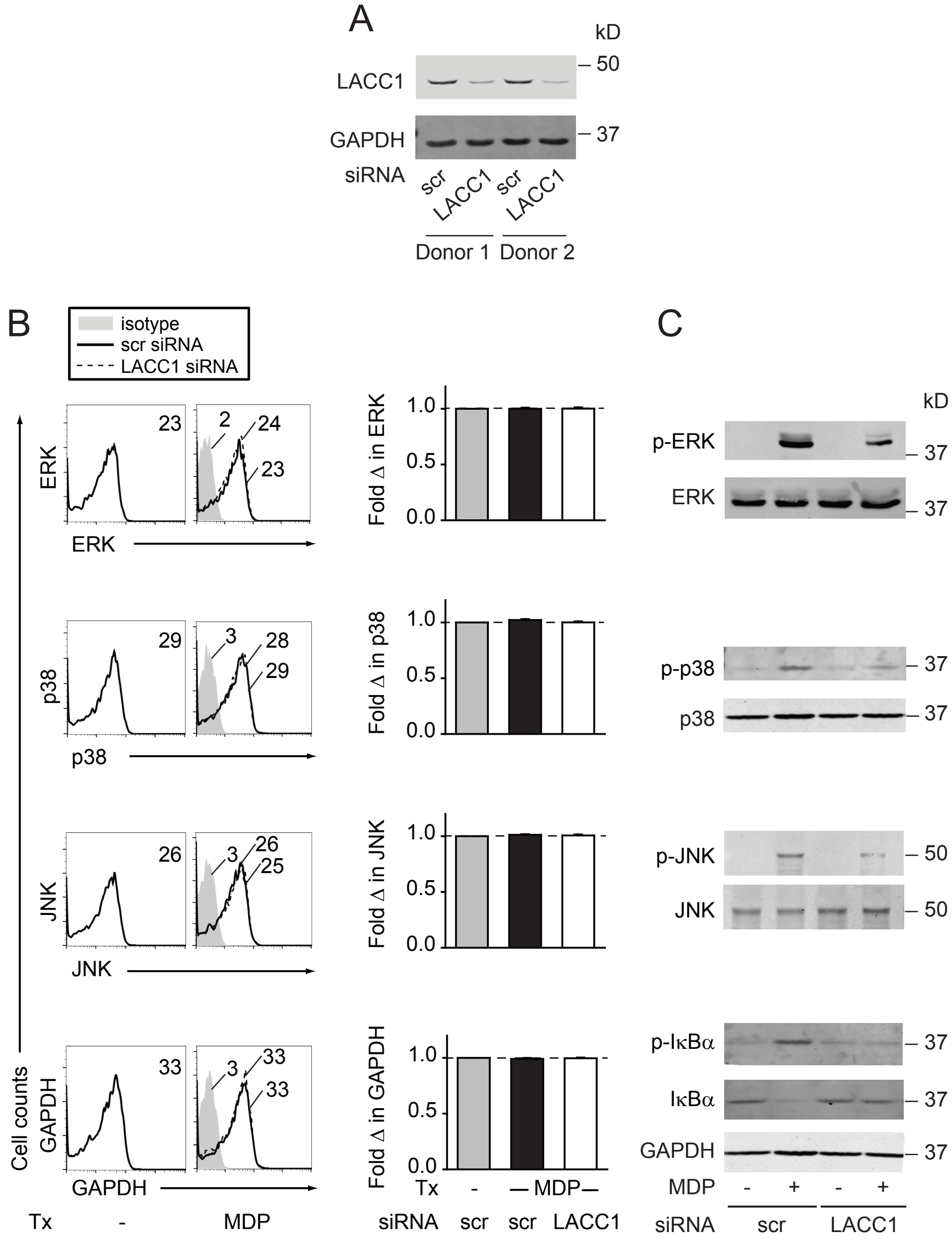

Supplementary Figure 5. LACC1 is required for optimal PRR-induced signaling. **(A)** A subset of MDMs from Figure 4A-B were simultaneously assessed for efficacy of LACC1 knockdown by Western blot. **(B-C)** MDMs were transfected with scrambled or LACC1 siRNA, then treated for 15min with 100 µg/ml MDP. **(B)** (*Left*) Representative flow cytometry plots with mean fluorescence intensity (MFI) values for ERK (Cell Signaling Technology, clone 137F5, diluted 1:1000), p38 (Santa Cruz Biotechnology, clone A-12, diluted 1:1000), JNK (Cell Signaling Technology, clone 56G8, diluted 1:1000) and GAPDH (n=8). (*Right)* Fold protein change normalized to untreated cells (represented by the dotted line at 1) + SEM. **(C)** Representative Western blot for phospho-ERK, phospho-p38, phospho-JNK and phospho-IκBα. Total protein for each is included (with antibodies as listed in '**B**', except JNK (Santa Cruz Biotechnology, clone D-2, diluted 1:1000) and IκBα (Cell Signaling Technology). As IκBα is degraded with activation, GAPDH is included as a control for equal loading. Tx, treatment; scr, scrambled.

A

MDP upregulated transcripts

	MDP					Curdlan		
	scr siRNA	LACC1 siRNA		MAPK NFκB inhibitors		scr siRNA	LACC1 siRNA	
Gene transcript	fold Δ	fold Δ	p value	fold Δ	p value	fold Δ	fold Δ	p value
TRAF1	10.0±0.30	2.41±0.29	1.70E-06	1.37±0.21	4.00E-07	1.25±0.22	1.13±0.20	NS
MARCSL1	9.91±1.46	2.44±0.19	2.26E-03	2.92±0.26	3.25E-03	1.14±0.14	1.28±0.25	NS
CSF2	9.83±0.92	2.17±0.48	3.23E-04	2.61±0.78	9.82E-04	1.09±0.24	1.27±0.25	NS
ICAM1	9.68±0.62	2.37±0.18	2.78E-05	1.28±0.19	1.27E-05	1.10±0.06	1.11±0.15	NS
A20	9.47±0.23	2.49±0.37	3.65E-06	1.47±0.28	5.85E-07	1.04±0.13	1.09±0.26	NS
IL1A	9.44±0.98	2.22±0.14	3.39E-04	1.28±0.14	1.72E-04	0.91±0.04	1.24±0.19	NS
IRG1	9.07±1.45	2.24±0.43	4.00E-03	2.46±0.85	7.63E-03	1.05±0.21	1.10±0.26	NS
RASL11A	9.02±0.71	1.04±0.10	3.17E-05	1.03±0.19	3.62E-05	0.97±0.13	1.13±0.16	NS
TLR2	8.61±0.89	4.15±0.50	4.77E-03	2.87±0.29	8.79E-04	1.09±0.12	1.12±0.15	NS
CDC42EP2	8.57±0.50	1.06±0.14	7.12E-06	0.98±0.13	6.45E-06	0.93±0.13	0.99±0.17	NS
IL6	8.41±0.88	1.97±0.20	3.72E-04	2.15±0.35	5.63E-04	0.94±0.11	1.02±0.17	NS
FSCN1	8.21±1.38	2.02±0.39	5.04E-03	2.53±0.37	7.43E-03	1.06±0.20	1.13±0.08	NS
SLC7A2	8.17±0.40	3.98±0.50	6.27E-04	2.18±0.27	1.70E-05	0.96±0.05	1.01±0.08	NS
NKX3-1	8.14±0.39	4.05±0.36	2.53E-04	1.18±0.06	2.14E-06	0.93±0.08	1.16±0.22	NS
GPR132	8.02±0.60	1.09±0.14	2.87E-05	1.12±0.18	3.24E-05	0.81±0.11	1.00±0.15	NS
OLR1	5.42±0.39	1.25±0.10	4.55E-05	1.40±0.21	9.89E-05	1.19±0.16	1.27±0.12	NS
SOCS3	5.12±0.36	1.12±0.09	3.87E-05	1.31±0.13	6.04E-05	1.08±0.13	1.23±0.09	NS
IL1B	4.97±0.47	0.99±0.08	1.58E-04	1.12±0.20	2.80E-04	1.18±0.12	1.26±0.22	NS
MMP14	4.84±0.37	2.56±0.44	7.11E-03	1.38±0.21	1.78E-04	1.10±0.15	1.22±0.11	NS
SLC27A4	4.84±0.50	1.02±0.14	3.17E-04	1.22±0.31	8.24E-04	1.12±0.19	1.19±0.10	NS
CD83	4.79±0.84	1.02±0.12	4.40E-03	1.13±0.13	5.09E-03	0.89±0.14	1.16±0.26	NS
SLC1A2	4.71±0.38	1.43±0.18	2.24E-04	1.32±0.34	5.58E-04	1.15±0.21	1.40±0.22	NS
S100A3	4.61±0.48	1.19±0.24	6.73E-04	1.40±0.27	1.11E-03	1.10±0.07	1.38±0.26	NS
MMP11	4.58±0.40	1.17±0.21	2.86E-04	1.27±0.33	6.90E-04	1.02±0.12	1.08±0.23	NS
STX11	4.44±0.46	1.17±0.18	5.68E-04	1.31±0.26	1.04E-03	1.03±0.14	1.20±0.22	NS
CD69	4.43±0.84	2.09±0.26	3.80E-02	2.07±0.38	4.31E-02	0.84±0.07	0.96±0.12	NS
IFIT1	4.33±0.32	1.03±0.13	8.02E-05	1.16±0.25	2.36E-04	0.91±0.16	0.98±0.20	NS
CD40	4.32±0.79	1.14±0.10	7.02E-03	1.09±0.12	6.70E-03	1.10±0.17	1.23±0.24	NS
MMP19	4.31±0.28	0.94±0.17	4.93E-05	1.14±0.23	1.27E-04	0.91±0.07	1.28±0.14	NS
ICOSL	4.26±0.41	1.81±0.18	1.66E-03	1.16±0.15	4.11E-04	0.95±0.09	1.14±0.12	NS
CKB	4.20±0.44	1.96±0.41	9.49E-03	2.40±0.37	1.94E-02	0.92±0.04	0.96±0.13	NS
SLC2A6	4.17±0.34	1.98±0.32	3.41E-03	2.27±0.26	4.29E-03	0.92±0.14	1.00±0.18	NS
TNF	3.69±0.89	1.02±0.07	2.39E-02	1.04±0.19	2.65E-02	0.91±0.15	1.22±0.16	NS
GPR84	3.52±0.36	0.82±0.06	3.35E-04	0.82±0.11	4.02E-04	0.75±0.08	0.99±0.18	NS

B

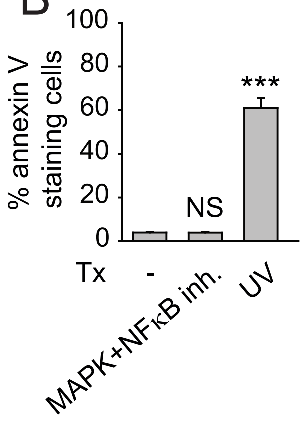

C

MDP and curdlan upregulated transcripts

	MDP					Curdlan		
	scr siRNA	LACC1 siRNA		MAPK NFκB inhibitors		scr siRNA	LACC1 siRNA	
Gene transcript	fold Δ	fold Δ	p value	fold Δ	p value	fold Δ	fold Δ	p value
CXCL1	10.1±1.00	2.41±0.10	2.59E-04	2.47±0.15	2.79E-04	8.11±0.49	11.3±1.60	NS
MMP12	8.85±0.79	1.12±0.17	7.48E-05	1.20±0.28	9.70E-05	9.41±1.11	10.2±1.00	NS
CXCL10	8.31±0.91	2.25±0.15	5.90E-04	1.32±0.30	7.12E-03	4.18±0.80	5.67±1.27	NS
RELB	7.98±0.83	4.12±0.47	6.68E-03	4.46±0.79	2.17E-02	4.55±0.82	4.92±0.90	NS
MMP10	7.47±0.55	2.13±0.33	1.68E-04	1.10±0.17	3.37E-05	4.08±0.35	4.15±0.53	NS
CCL5	7.06±0.12	2.11±0.10	7.06E-08	2.19±0.18	5.13E-07	3.91±0.23	4.75±0.81	NS
NFKB2	4.80±0.66	1.16±0.13	1.64E-03	1.24±0.26	2.42E-03	2.28±0.28	2.46±0.36	NS
NFKBIZ	4.74±0.51	0.97±0.15	3.88E-04	1.02±0.13	3.89E-04	8.34±0.78	10.4±1.17	NS
CXCL2	4.35±0.25	2.27±0.26	1.15E-03	1.36±0.18	2.39E-04	8.70±0.99	9.20±1.53	NS
NFKBIE	4.20±0.53	2.01±0.37	1.49E-02	1.16±0.16	1.57E-03	1.91±0.27	2.27±0.51	NS
TNFAIP3	4.19±0.32	2.20±0.35	5.81E-03	1.17±0.19	1.94E-04	3.96±0.75	4.21±0.87	NS
REL	4.17±0.87	0.87±0.04	9.03E-03	1.14±0.13	1.36E-02	3.49±0.67	4.43±0.88	NS
CXCL11	4.11±0.11	1.16±0.12	1.65E-06	1.22±0.11	8.05E-06	3.93±0.21	4.43±0.63	NS
PDE4B	3.85±0.40	1.72±0.33	5.88E-03	1.70±0.15	2.23E-06	1.64±0.26	2.05±0.34	NS
CSF1	2.41±0.05	1.05±0.17	2.77E-04	1.17±0.23	1.92E-03	4.62±0.39	4.73±0.52	NS

D

curdlan upregulated transcripts

	MDP					Curdlan		
	scr siRNA	LACC1 siRNA		MAPK NFκB inhibitors		scr siRNA	LACC1 siRNA	
Gene transcript	fold Δ	fold Δ	p value	fold Δ	p value	fold Δ	fold Δ	p value
EGR1	0.99±0.09	1.04±0.16	NS	1.16±0.14	NS	9.55±1.35	11.6±2.31	NS
MCLON3	1.25±0.19	0.97±0.09	NS	1.09±0.12	NS	8.49±0.91	10.9±2.92	NS
EGR2	1.05±0.15	0.80±0.16	NS	1.03±0.14	NS	8.39±1.18	8.27±1.44	NS
MPO	0.96±0.11	0.90±0.07	NS	0.84±0.14	NS	8.33±1.31	7.26±1.12	NS
PPAP2B	0.91±0.07	0.96±0.08	NS	0.96±0.08	NS	8.07±0.65	9.23±0.66	NS
PRDM1	1.08±0.09	0.97±0.11	NS	1.29±0.06	NS	5.02±0.55	5.19±0.26	NS
HSPA1A	0.87±0.03	0.96±0.12	NS	1.19±0.20	NS	4.73±0.46	4.99±0.47	NS
EGR3	0.83±0.07	0.83±0.10	NS	1.13±0.25	NS	4.64±0.49	5.35±1.22	NS
THBS1	0.92±0.12	0.98±0.05	NS	1.22±0.21	NS	4.56±0.41	5.16±0.37	NS
F5	0.93±0.12	0.82±0.12	NS	1.12±0.13	NS	3.90±0.80	4.74±0.84	NS
FABP4	0.92±0.10	0.92±0.17	NS	1.26±0.27	NS	3.82±0.34	4.12±0.59	NS
ADAMTS1	1.05±0.11	0.89±0.11	NS	1.27±0.22	NS	3.41±0.26	4.22±0.33	NS
HSPA1B	1.05±0.08	1.08±0.15	NS	1.50±0.23	NS	2.46±0.25	2.83±0.24	NS

Supplementary Figure 6. LACC1 is required for optimal expression of a broad range of NOD2-induced transcripts. MDMs (n=4) were transfected with scrambled or LACC1 siRNA, or pretreated for 1 h with 0.1 μM JNK inhibitor II, PD98059 (ERK inhibitor), SB202190 (p38 inhibitor) and BAY 11-7082 (NFκB inhibitor) (EMD Biosciences), then treated with 100μg/ml MDP (NOD2 ligand) or 100μg/ml curdlan (dectin ligand) for 4h. mRNA at 4h is normalized to scrambled siRNA-transfected, untreated cells. Fold mRNA change of indicated transcripts is classified according to: **(A)** MDP-upregulated transcripts, **(C)** MDP- and curdlan-upregulated transcripts, and **(D)** curdlan-upregulated transcripts. Significance is compared to scrambled siRNA-transfected, MDP-treated cells. **(B)** MDMs (n=4) were treated for 4h with 0.1 μM JNK inhibitor II, PD98059 (ERK inhibitor), SB202190 (p38 inhibitor) and BAY 11-7082 (NFκB inhibitor). Cell death was assessed by annexin V and shown as percent annexin V^+ cells + SEM. 50-100 J/m^2 UV-treated cells is shown as a positive control. Tx, treatment; inh., inhibitors; NS, not significant. ***, p<0.001.

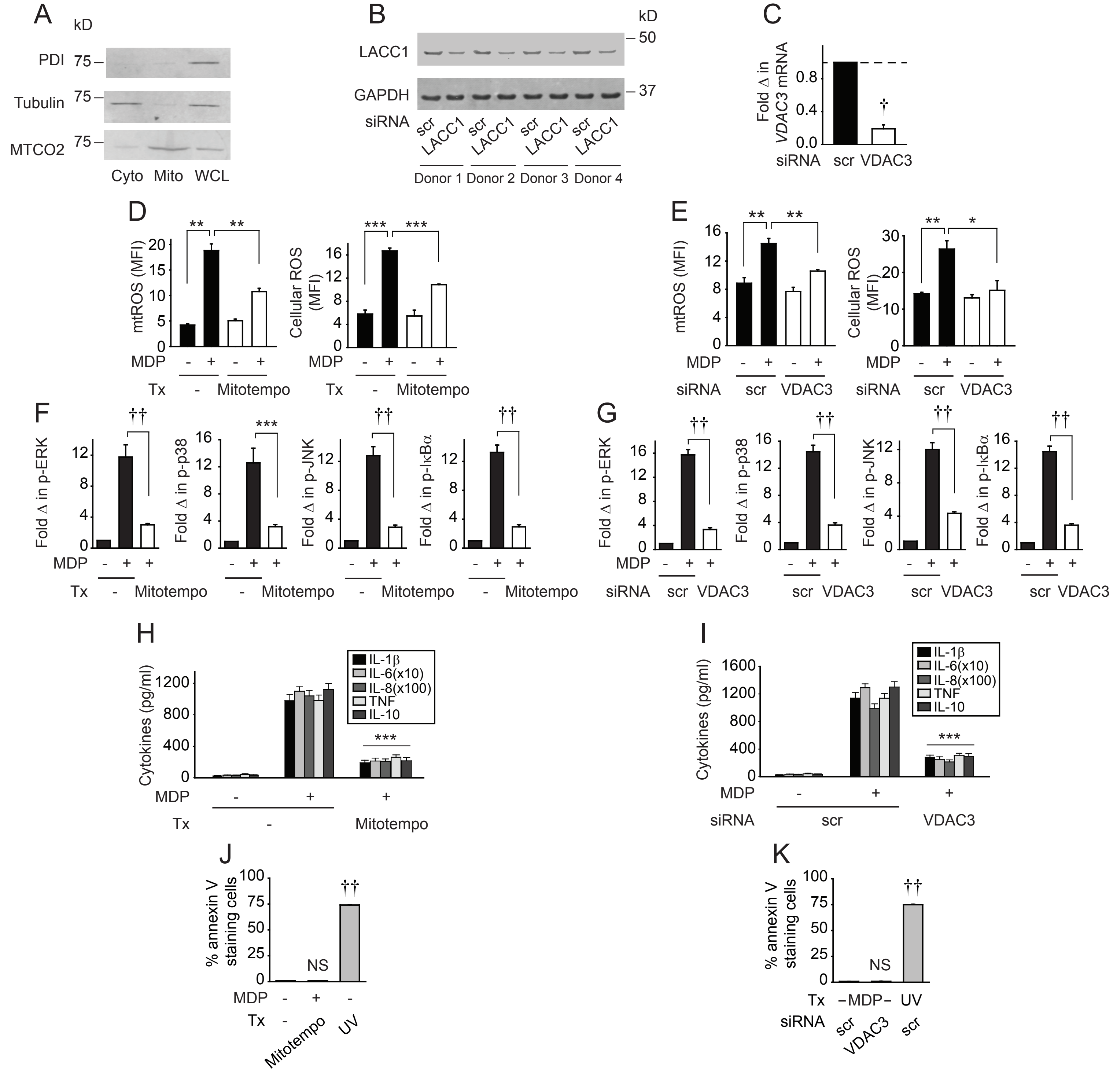

Supplementary Figure 7. MtROS contributes to NOD2-induced signaling and cytokine secretion in MDMs. (A) MDMs were fractionated into cytoplasmic (cyto) or mitochondrial (mito) compartments and assessed for PDI (Cell Signaling Technology, clone C81H6, diluted 1:1000) (ER marker) expression. Shown is a representative Western blot. Tubulin and MTCO2 were used as cytoplasmic and mitochondrial controls, respectively. Whole cell lysate (WCL) is included as a control for all markers assessed. **(B)** A subset of MDMs from Figure 4D-E was simultaneously assessed for efficacy of LACC1 knockdown by Western blot. **(C)** MDMs were transfected with scrambled or VDAC3 siRNA. *VDAC3* mRNA expression normalized to scrambled siRNA-transfected cells + SEM (n=4). **(D,F,H,J)** MDMs were pre-incubated with the mtROS inhibitor Mito-Tempo (50nM, Santa Cruz Biotechnology) for 20min, then treated with 100 μg/ml MDP and assessed for: **(D)** mtROS (n=4) and cellular ROS (n=4) at 6h, **(F)** phospho-protein induction normalized to untreated cells at 15 min as assessed by flow cytometry, **(H)** cytokines at 24h (n=4), and **(J)** cell death (n=4) as assessed by annexin V with 50-100 J/m^2 UV-treated cells shown as a positive control. **(E,G,I,K)** MDMs were transfected with scrambled or VDAC3 siRNA, then treated with 100 μg/ml MDP and assessed for: **(E)** mtROS and ROS at 6h (n=4), **(G)** phospho-protein induction normalized to untreated cells at 15 min as assessed by flow cytomery, **(I)** cytokines at 24h (n=4) and **(K)** cell death at 24h (n=4). Mean + SEM for '**C-K**'. NS, not significant; scr, scrambled; tx, treatment. *, $p<0.05$; **, $p<0.01$; ***, $p<0.001$; †, $p<1x10^{-4}$; ††, $p<1x10^{-5}$.

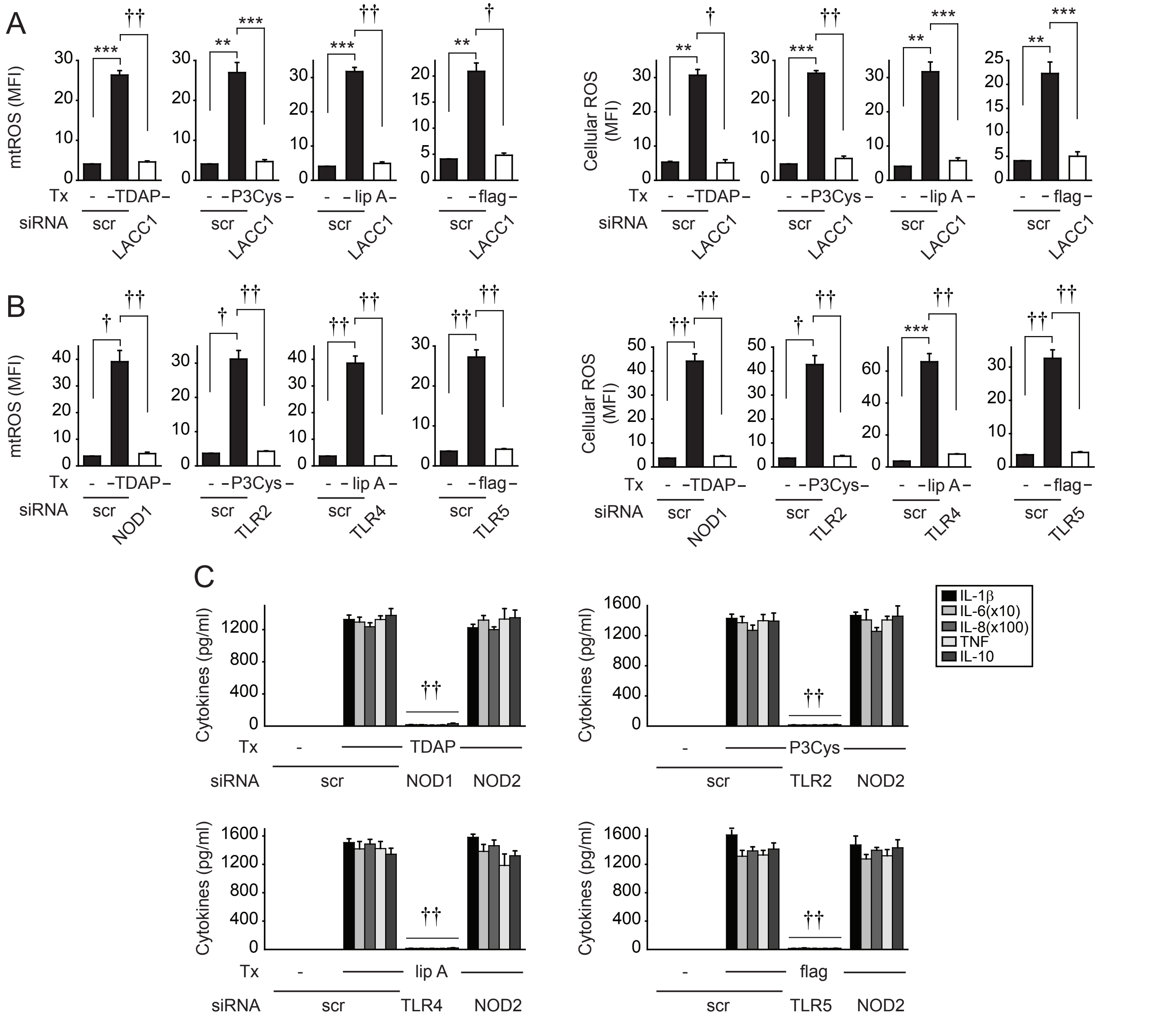

Supplementary Figure 8. LACC1 is required for optimal mtROS and cellular ROS upon stimulation of multiple PRRs. (A) MDMs (n=4) were transfected with scrambled or LACC1 siRNA, then treated with 100 μg/ml TriDAP (NOD1 ligand; TDAP), 10 μg/ml Pam3Cys (TLR2 ligand; P3Cys), 0.1 μg/ml lipid A (TLR4 ligand; lip A), or 5 ng/ml flagellin (TLR5 ligand; flag) and assessed for mtROS and cellular ROS at 6h (n=4). **(B-C)** MDMs (n=4) were transfected with scrambled, NOD1, TLR2, TLR4 or TLR5 siRNA, then treated with 100 μg/ml TriDAP, 10 μg/ml Pam3Cys, 0.1 μg/ml lipid A, or 5 ng/ml flagellin and assessed for: **(B)** mtROS and cellular ROS at 6h, or **(C)** cytokines at 24h. As a control, MDMs in '**C**' were also transfected with siRNA to NOD2 to demonstrate responsivity of the cells to the non-NOD2 ligands. Scr, scrambled; tx, treatment. **, p<0.01; ***, p<0.001; †, p<1x10^{-4}; ††, p<1x10^{-5}.

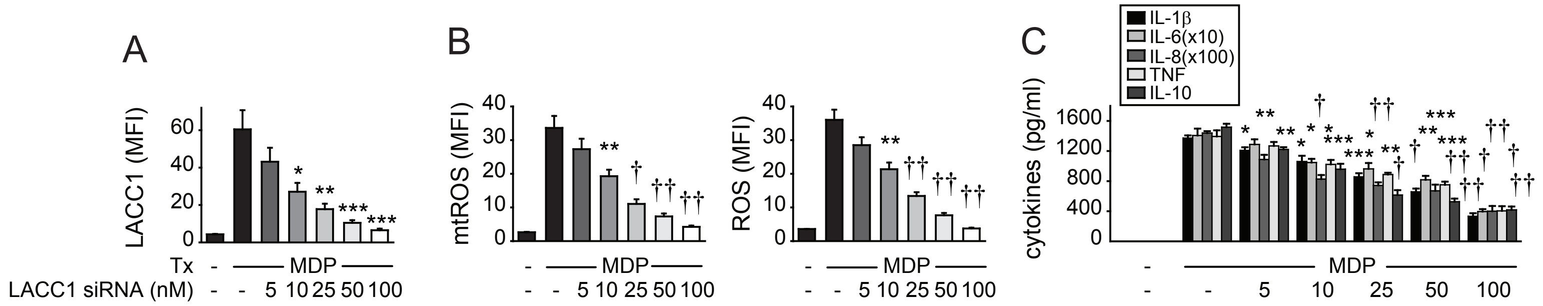

D

	MDP										
	scr siRNA	LACC1 siRNA									
		5 nM		10 nM		25 nM		50 nM		100 nM	
Gene transcript	fold Δ	fold Δ	p value	fold Δ	p value	fold Δ	p value	fold Δ	p value	fold Δ	p value
TRAF1	7.39±0.55	7.38±1.19	NS	4.05±0.70	9.67E-03	2.66±0.51	7.46E-04	2.12±0.24	1.23E-04	1.70±0.32	7.56E-05
MARCKSL1	9.69±1.23	6.55±1.03	NS	5.60±0.69	2.71E-02	3.10±0.43	2.33E-03	2.60±0.21	1.28E-03	1.56±0.24	5.99E-04
CSF2	7.75±1.36	5.20±0.73	NS	4.25±0.51	NS	2.23±0.28	7.24E-03	2.58±0.71	1.49E-02	1.28±0.19	3.93E-03
ICAM1	9.32±1.15	5.51±0.54	2.38E-02	4.26±0.41	6.07E-03	2.26±0.17	9.06E-04	1.54±0.27	5.94E-04	1.23±0.27	4.00E-04
A20	9.64±1.48	5.96±0.50	NS	3.72±0.85	1.33E-02	2.08±0.47	2.83E-03	1.66±0.35	1.94E-03	1.51±0.19	1.38E-03
IL1A	8.13±1.33	5.27±0.63	NS	4.22±0.49	3.32E-02	2.26±0.34	5.23E-03	1.98±0.16	3.75E-03	1.10±0.15	1.92E-03
IRG1	7.87±1.48	5.10±0.82	NS	4.25±0.46	NS	2.24±0.20	9.17E-03	1.93±0.16	7.11E-03	1.10±0.15	3.65E-03
RASL11A	8.83±1.26	5.70±0.80	NS	4.48±0.67	2.26E-02	2.37±0.28	2.46E-03	1.79±0.33	1.67E-03	0.56±0.11	6.00E-04
TLR2	10.1±1.71	9.58±2.04	NS	7.22±1.04	NS	5.11±0.88	4.11E-02	4.40±0.67	2.10E-02	2.85±0.60	5.35E-03
CDC42EP2	8.53±1.20	5.29±0.68	NS	4.20±0.56	1.69E-02	2.31±0.30	2.37E-03	1.84±0.10	1.43E-03	0.82±0.13	6.44E-04

Supplementary Figure 9. The level of LACC1 expression regulates NOD2-induced mtROS and ROS production, cytokine secretion and transcript expression. MDMs were transfected with scrambled or LACC1 siRNA at the indicated concentrations, then treated with 100 µg/ml MDP and assessed for: **(A)** LACC1 protein expression at 12h as assessed by flow cytometry (n=8), **(B)** mtROS and cellular ROS at 6h (n=8), **(C)** cytokines at 24h (n=4) and **(D)** mRNA induction of the top 10 upregulated transcripts from Fig S6A at 4h normalized to scrambled siRNA-transfected, untreated cells (n=4); significance is compared to scrambled siRNA-transfected, MDP-treated cells. Tx, treatment, scr, scrambled; *, $p<0.05$; **, $p<0.01$; ***, $p<0.001$; †, $p<1x10^{-4}$; ††, $p<1x10^{-5}$.

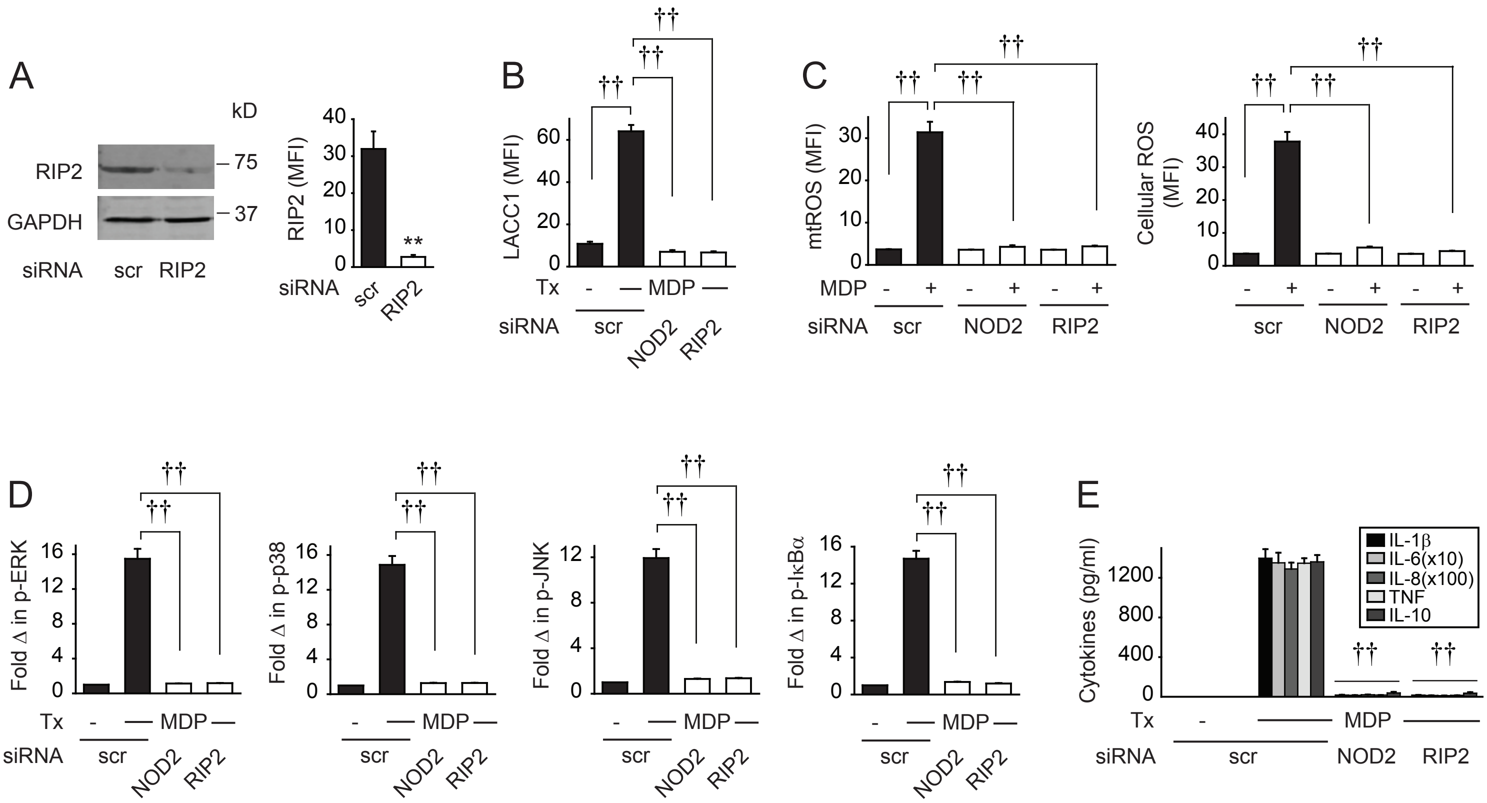

Supplementary Figure 10. NOD2 and RIP2 are required for MDP-mediated upregulation of LACC1 expression, mtROS and ROS production, signaling and cytokine secretion. MDMs were transfected with scrambled, NOD2, or RIP2 siRNA, then **(A)** left untreated and assessed for RIP2 protein expression by (*left*) Western blot and (*right)* flow cytometry expressed as MFI + SEM (n=4) or **(B-E)** treated with 100 µg/ml MDP and assessed for: **(B)** LACC1 protein expression by flow cytometry and expressed as MFI at 12h (n=8), **(C)** mtROS and cellular ROS at 6h (n=8), **(D)** phospho-protein induction normalized to untreated, scrambled siRNA-transfected cells at 15 min (n=8) as assessed by flow cytometry and **(E)** cytokines at 24h (n=4). Mean + SEM for '**B-E**'. Tx, treatment, scr, scrambled; **, p<0.01; ††, p<1x10^{-5}.

A

Kingdom	Species	Gene bank accession number	Identity to human LACC1	Identity to human laccase domain (aa* 195-427) (AKA* Cu-Oxidase-4 domain)
Mammalia	*Homo sapiens*	NP_001121775		
	Canis lupus	XP_005633973	84.9%	91.4%
	Bos taurus	NP_001193037	84.9%	88.8%
	Mus musculus	NP_766076	77.2%	85.0%
	Rattus norvegicus	XP_233749	75.8%	86.7%
Aves	*Gallus gallus*	XP_417036	60.6%	75.6%
Teleosts	*Danio rerio*	XP_001919421	50.4%	60.6%
Mollusks	*Crassostrea gigas*	EKC22555	42.3%	43.6%
Bacteria	*Paenibacillus sp*	EES72500	33.6%	33.2%

*aa, amino acid; AKA, also known as

B

Homo sapiens (Human)	234 NAAGFNVEKFYRIKTHHSNDIWIMGRKEPDS 264
Canis lupus (Dog)	NAAGFNVEKFYRIKTDHANDVWIMGRKEPES
Bos taurus (Cow)	NAAGFNVEKFYQIKTDHANDVWIMGRKEPES
Mus musculus (Mouse)	NAAGFNAEKFYRIKTDHASEVWVMGKKEPES
Rattus norvegicus (Rat)	NAAGFNAEKFYRIKTDHASEVWVMGKKEPES
Gallus gallus (Chicken)	NAAGFNPENFHRVKTDHANAVCVMGRTEPDS
Danio rerio (Zebrafish)	.QAGFHSRQLNLIKCNHASDVWVMGKPAPDS
Crassostrea gigas (Oyster)	SKAGFDVKTLYIAKAVHGNTVYEIGTDPPDG
Paenibacillus sp (Bacteria)	EALGFAPEAWTCGEQVHANAVAVVRAEDRGK

C

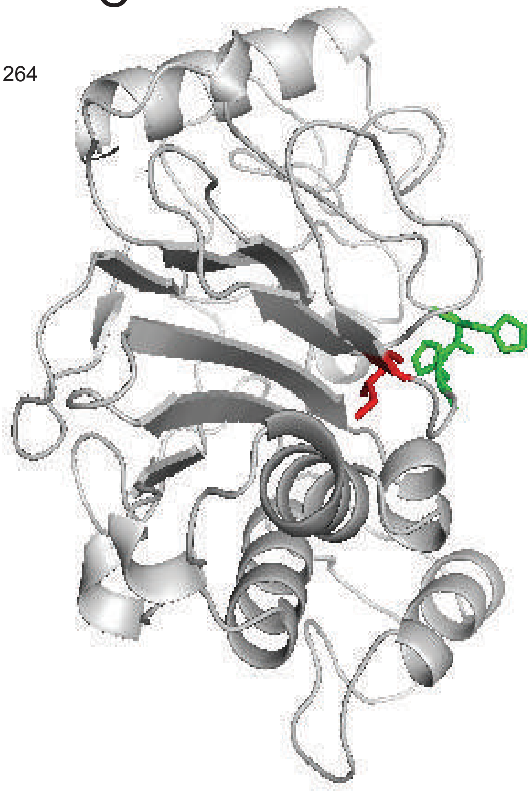

Supplementary Figure 11. LACC1 protein identity and LACC1 variants. (A) Table showing identity for human full-length LACC1 and the laccase domain-only component relative to other species. **(B)** Comparison of the region encompassing the Ile254 and His249,250 amino acids within the laccase-domain across species to demonstrate degree of conservation of these amino acids. Color coding within rectangles highlights the amino acids under consideration and correlate to the colors shown in the predicted structure model in '**C**'. **(C)** Model of LACC1 structure generated per Swiss Model with the Ile254 (red) and His249 and His250 amino acids (green) highlighted in the overall structure, thereby demonstrating proximity to each other in a putative metal binding pocket.

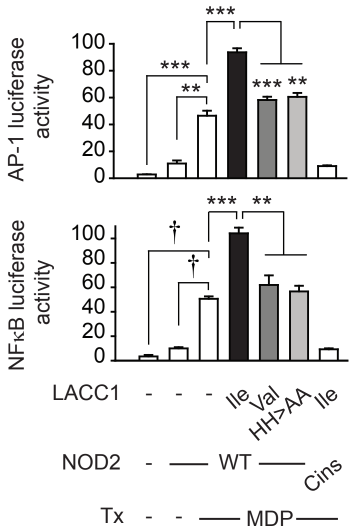

Supplementary Figure 12. Transfection of the LACC1 risk variant results in decreased NOD2-induced AP-1 and NFκB transcriptional activity upon low dose MDP treatment. Empty vector, LACC1 Ile254 and Val254 variants, or LACC1 His249,250Ala mutants were transfected into HEK293 cells along with NOD2 ± AP-1 or NFκB luciferase and Renilla constructs. Transfected cells were treated with 10 μg/ml MDP and assessed for AP-1 and NFκB luciferase activity at 6h. Included is also the Crohn's disease-associated NOD2 LeufsinsC (Cins) variant cotransfected with LACC1 Ile254 as a control for the specificity of NOD2 responsitivity to MDP treatment. Represented is mean + SEM for 3 replicates. Tx, treatment. **, $p<0.01$; ***, $p<0.001$; †, $p<1x10^{-4}$.

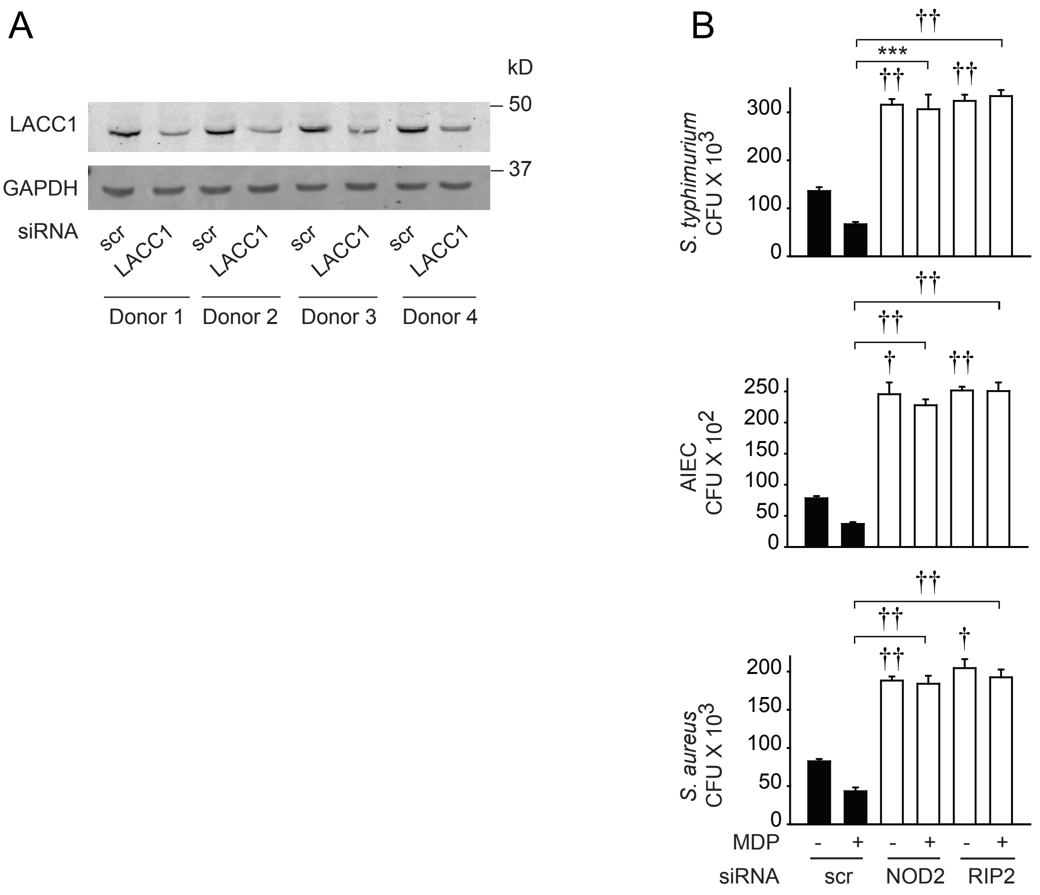

Supplementary Figure 13. NOD2 and RIP2 are required for optimal MDP-induced intracellular microbial clearance. **(A)** A subset of MDMs from Figure 6B was simultaneously assessed for efficacy of LACC1 knockdown by Western blot. **(B)** MDMs (n=4) were transfected with scrambled, NOD2 or RIP2 siRNA, then treated with 100 µg/ml MDP for 48h, followed by co-culture with *S. typhimurium*, AIEC or *S. aureus*. Clearance of intracellular bacteria is represented as colony forming units (CFU) + SEM. Significance is compared to untreated, scrambled siRNA-transfected MDMs or as indicated. Scr, scrambled. ***, $p<0.001$; †, $p<1x10^{-4}$; ††, $p<1x10^{-5}$.

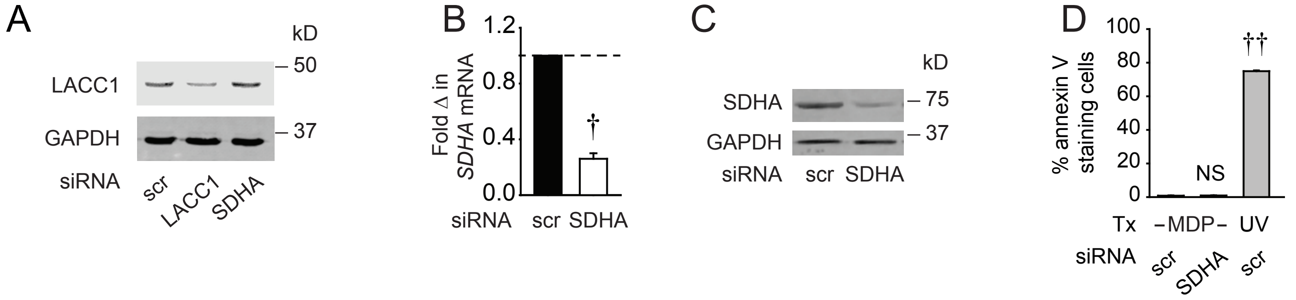

Supplementary Figure 14. SDHA knockdown does not affect the viability of MDMs. (A) A subset of MDMs from Figure 7B was simultaneously assessed for efficacy of LACC1 knockdown by Western blot (representative 1 of 4 donors). Both scrambled and SDHA siRNA serve as negative controls. **(B-D)** MDMs were transfected with scrambled or SDHA siRNA, then treated with 100 µg/ml MDP for 2h and assessed for: knockdown efficiency by **(B)** mRNA (n=4) and **(C)** protein as assessed by Western blot, or **(D)** cell death (n=4) as assessed by annexin V with 50-100 J/m^2 UV-treated cells shown as a positive control. Mean + SEM for '**B**' and '**D**'. Scr, scrambled; Tx, treatment; NS, not significant. †, $p<1x10^{-4}$; ††, $p<1x10^{-5}$.

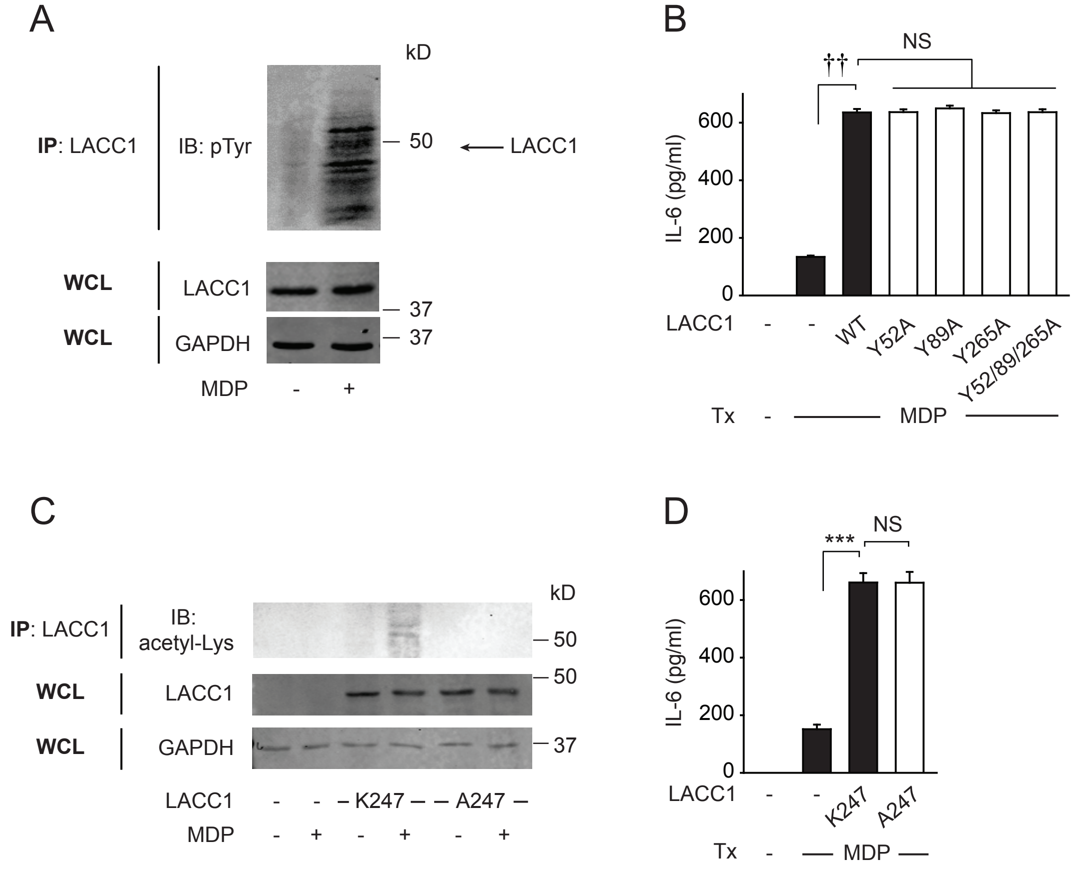

Supplementary Figure 15. Mutation of select tyrosines or of a putative acetylated lysine in LACC1 does not alter NOD2-induced cytokines. (A) LACC1 was immunoprecipitated (IP) from cell lysates and tyrosine phosphorylation (EMD Millipore, clone 4G10, diluted 1:1000) was assessed by Western blot (IB) at 15 min. LACC1 and GAPDH were used as loading controls. **(B)** Empty vector, WT LACC1 (Ile254), LACC1 Tyr52Ala, Tyr89Ala, Tyr265Ala or Tyr52,89,265Ala mutants were transfected into HEK293 cells along with NOD2 and then treated with 100 μg/ml MDP for 24h. IL-6 secretion was assessed. **(C-D)** Empty vector, WT LACC1 (Lys247) or LACC1 Ala247 were transfected into HEK293 cells along with NOD2 and then treated with 100 μg/ml MDP. **(C)** LACC1 was immunoprecipitated (IP) from cell lysates and acetylation (Abcam) was assessed at 15 min by Western blot. LACC1 and GAPDH were used as controls. **(D)** IL-6 secretion was assessed at 24h. Mean + SEM for '**B**' and '**D**'. Tx, treatment; WCL, whole cell lysate; NS, not significant. ***, $p<0.001$; ††, $p<1x10^{-5}$.

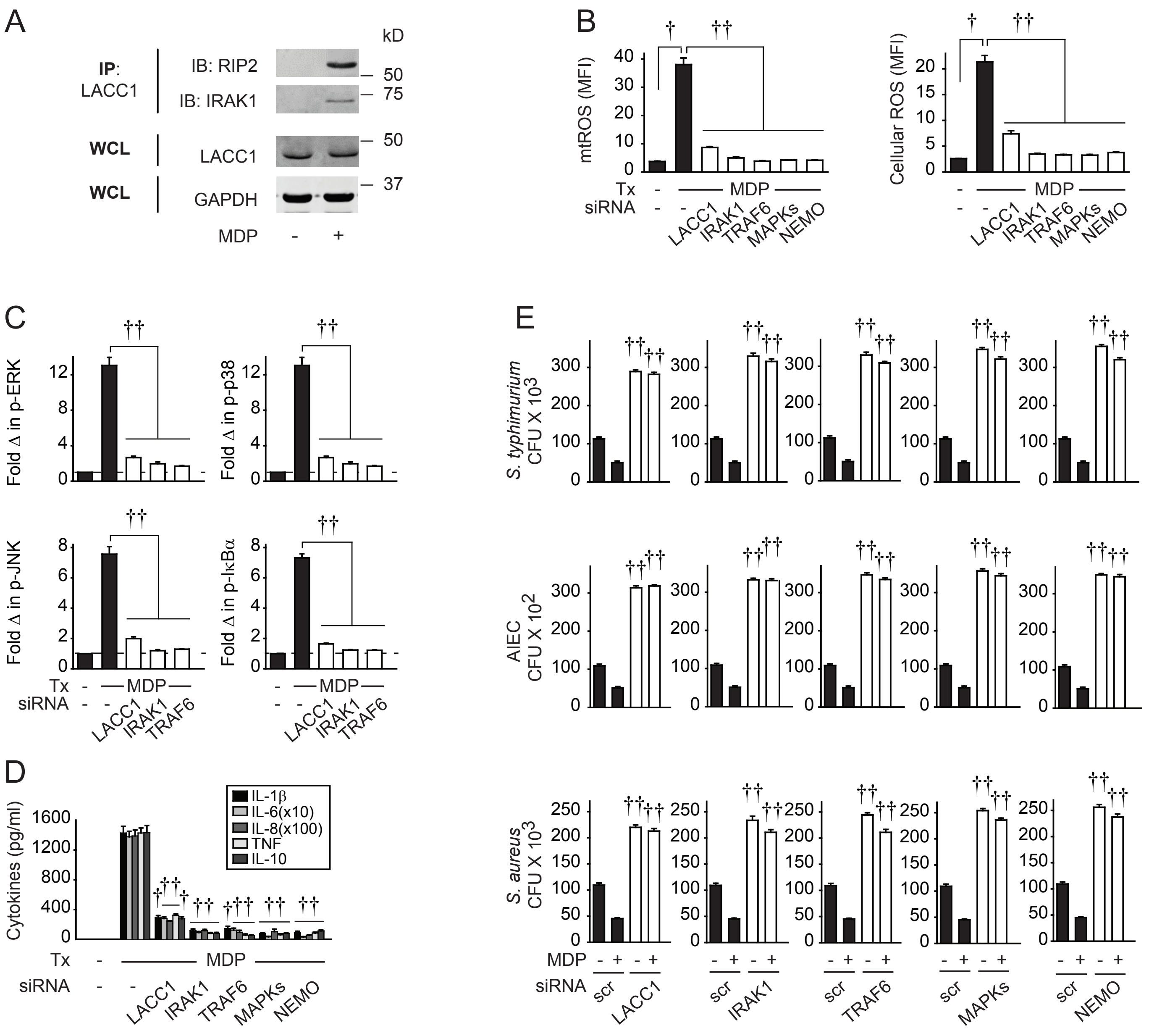

Supplementary Figure 16. LACC1 assembles in a complex with NOD2 pathway intermediates and these intermediates are required for LACC1-dependent outcomes. (A) MDMs were treated with 100 μg/ml MDP for 15 min. LACC1 was immunoprecipitated (IP) from cell lysates and the recruitment of the indicated proteins was assessed by Western blot (IB). LACC1 and GAPDH were used as loading controls. **(B-E)** MDMs were transfected with scrambled or the indicated siRNA, then treated with 100 μg/ml MDP and assessed for: **(B)** mtROS and cellular ROS at 6h (n=6), (**C**) phospho-protein induction normalized to scrambled siRNA-transfected cells at 15 min (n=8) as assessed by flow cytometry, **(D)** cytokine secretion at 24h (n=4), and **(E)** intracellular bacterial clearance at 48h (n=4; significance is compared with the corresponding scrambled siRNA condition). Mean + SEM for '**B-E**'. Tx, treatment; MAPKs (ERK, p38 and JNK); scr, scrambled; WCL, whole cell lysate. †, $p<1x10^{-4}$; ††, $p<1x10^{-5}$.

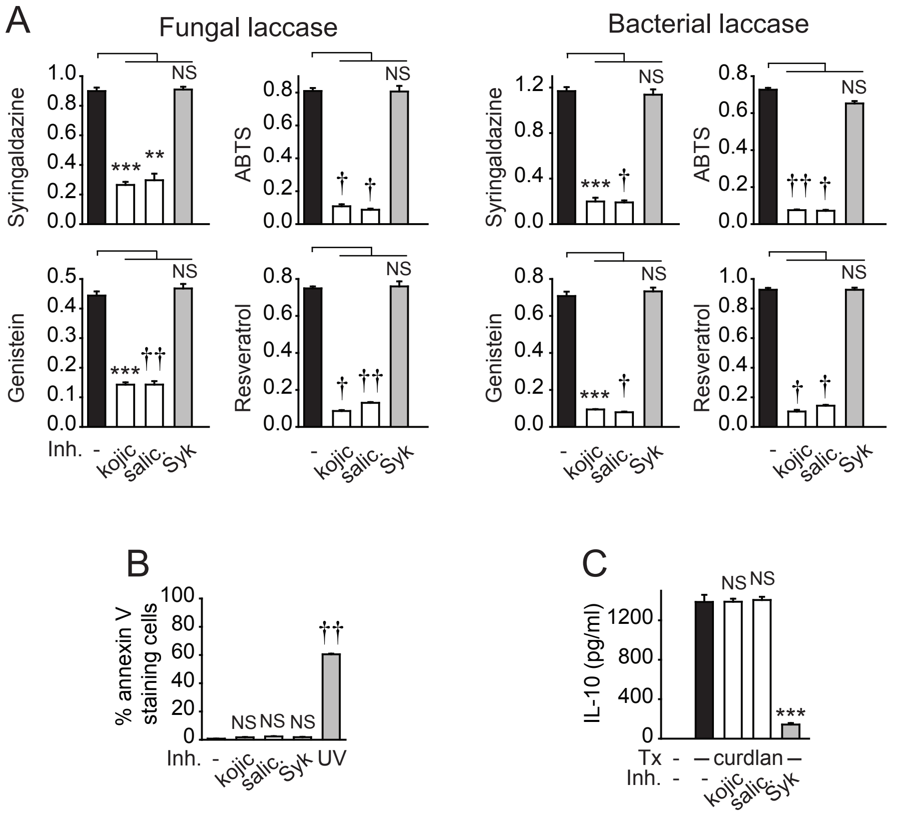

Supplementary Figure 17. Polyphenol oxidase activity is diminished with select inhibitors in vitro. **(A)** *Rhus vernicifera* laccase (fungal laccase) (2 µg/ml) (*left*) or *E. coli* CueO laccase (bacterial laccase) (2 µg/ml) (*right*) were preincubated with kojic acid, salicylhydroxamic acid (polyphenol oxidase inhibitors) or a Syk inhibitor for 1h, then incubated with the indicated polyphenol oxidase substrates and assessed for oxidation. Data represent 4 replicates. **(B-C)** MDMs were preincubated with kojic acid, salicylhydroxamic acid or a Syk inhibitor for 1h, and then assessed for: **(B)** cell death at 24 h or **(C)** IL-10 secretion following treatment with 100 µg/ml curdlan for 24h. Mean + SEM for '**A-C**'. Tx, treatment; inh, inhibitors; salic, salicylhydroxamic acid. NS, not significant. **, $p<0.01$; ***, $p<0.001$; †, $p<1x10^{-4}$; ††, $p<1x10^{-5}$.

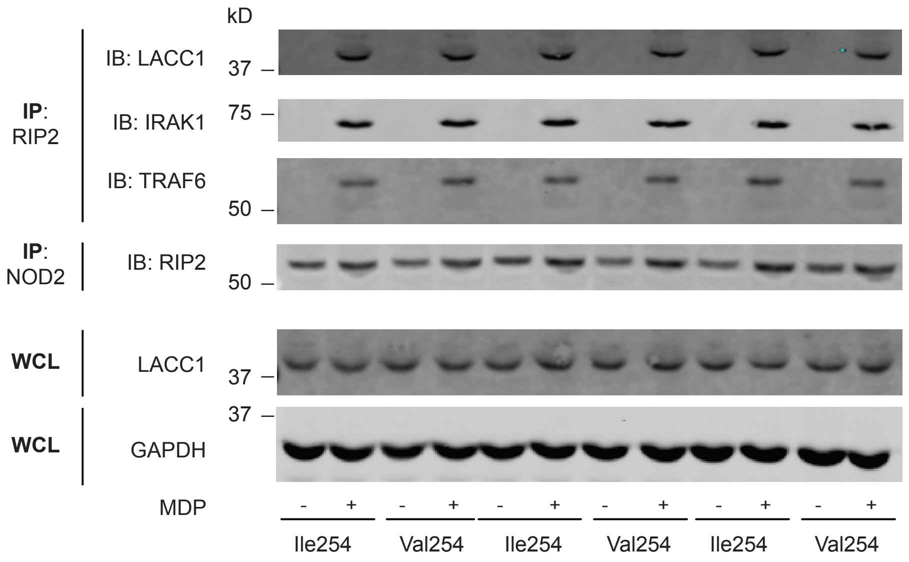

Supplementary Figure 18. MDMs from LACC1 Val254 risk carriers show similar recruitment of NOD2 pathway intermediates relative to LACC1 Ile254 carriers. MDMs from LACC1 Ile254 and Val254 carriers (n=11-12 per genotype) were treated with 100 μg/ml MDP for 15 min. RIP2 or NOD2 were immunoprecipitated (IP) from cell lysates and the recruitment of the indicated proteins was assessed by Western blot (IB). Shown is a representative Western blot for n=3/genotype. LACC1 and GAPDH were assessed as loading controls. WCL, whole cell lysate.

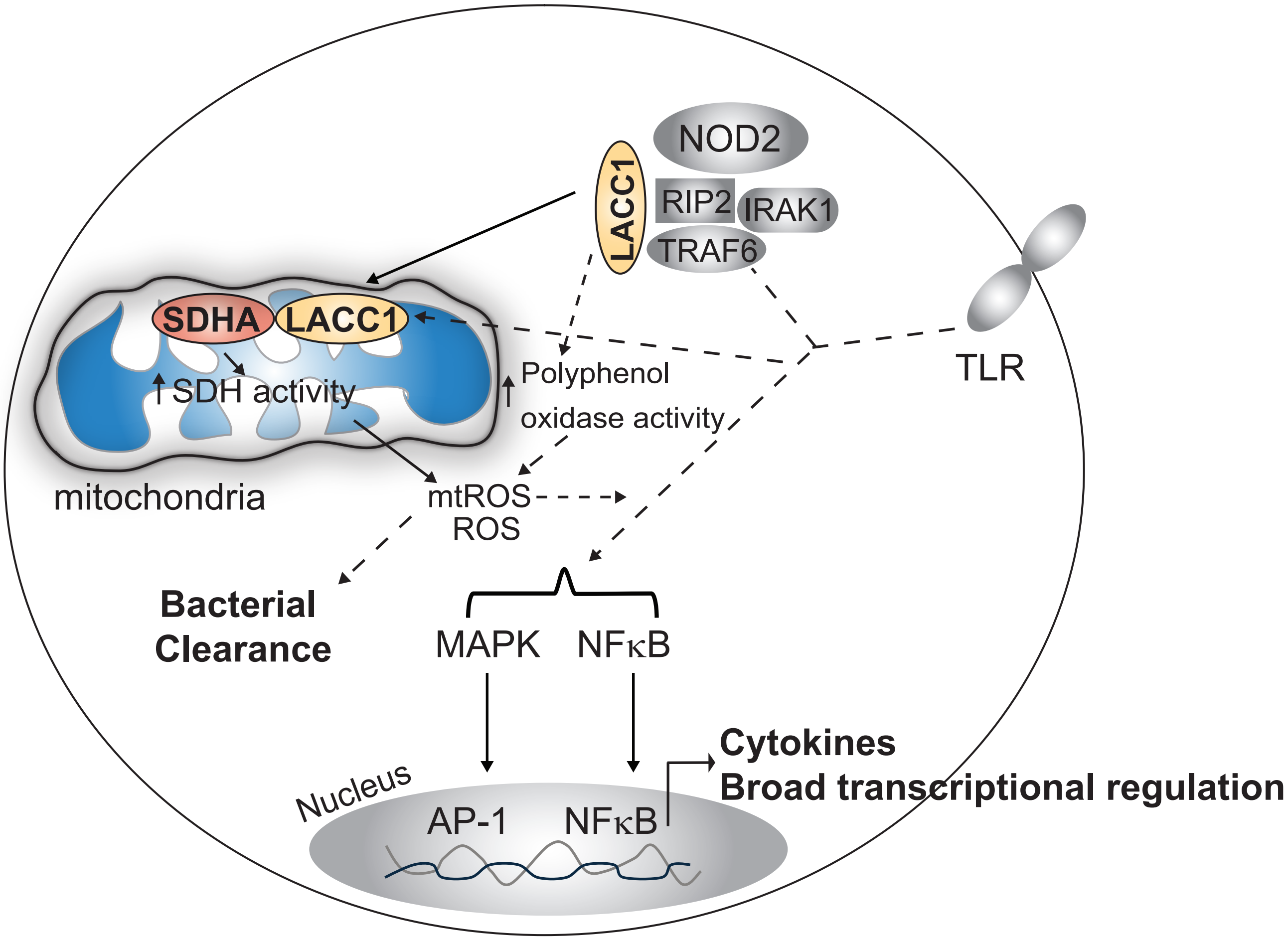

Supplementary Figure 19. A model for the role of LACC1 in amplifying PRR-induced pathways. Upon NOD2 stimulation, LACC1 associates with a complex that contains NOD2 and the proximal signaling intermediates, RIP2, IRAK1 and TRAF6. LACC1 is, in turn, required for the polyphenol oxidase activity, mtROS and cellular ROS production, MAPK and NFκB pathway activation, and subsequent cytokine secretion and intracellular bacterial clearance that occurs upon NOD2 stimulation. LACC1 also localizes to mitochondria and associates with SDHA. Upon NOD2 stimulation, LACC1 enhances SDH activity, which in turn, is required for each of the NOD2-induced downstream outcomes. Importantly, LACC1 is required for optimal cytokine secretion upon stimulation with a broad range of PRR ligands and mycobacterial antigens. Relative to the LACC1 Ile254 variant, NOD2-induced polyphenol oxidase activity, mtROS and cellular ROS production, MAPK and NFκB pathway activation, and subsequent cytokine secretion are lower in cells transfected with the Crohn's disease risk LACC1 Val254 variant or with mutation of the nearby LACC1 His249,250. Similarly, MDMs from LACC1 Val254 risk carriers demonstrate decreased NOD2-induced polyphenol oxidase activity, mtROS and ROS production, signaling, cytokine secretion and bacterial clearance.

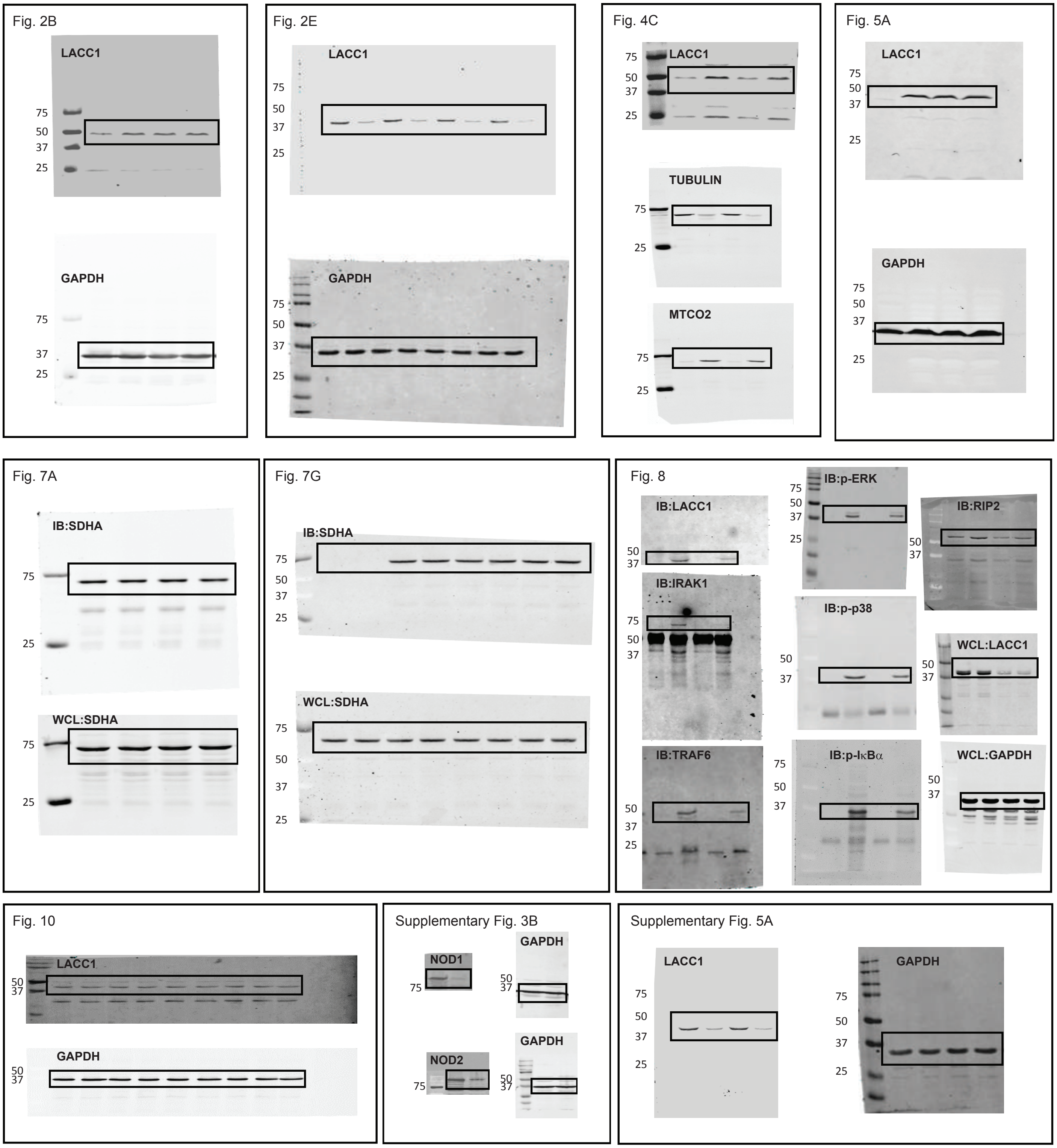

Supplementary Figure 20. Western blots for manuscript.

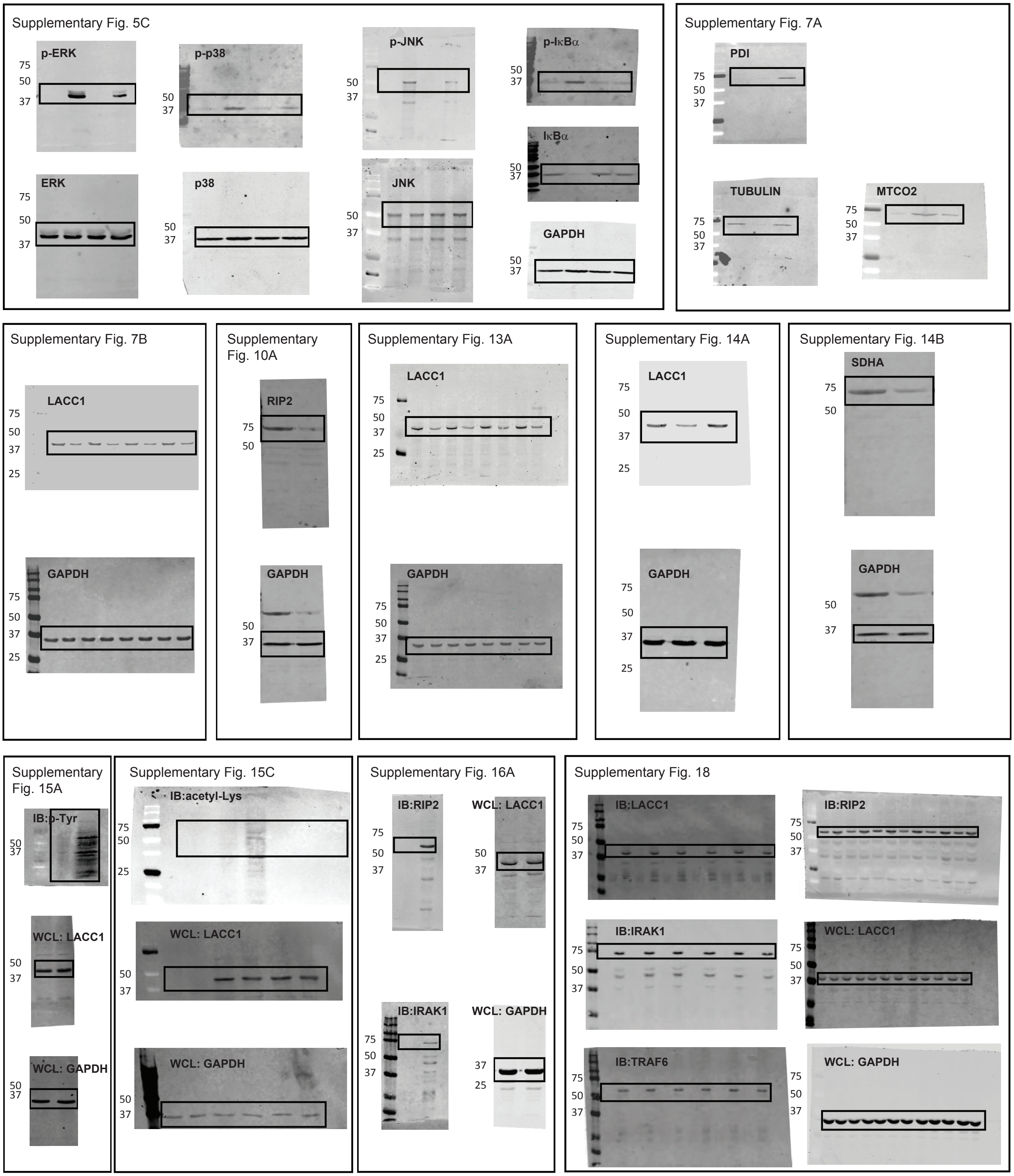

Supplementary Fig. 5C
p-ERK
75
50
37
ERK
75
50
37
p-p38
50
37
p38
50
37
p-JNK
50
37
JNK
50
37
p-IκBα
50
37
IκBα
50
37
GAPDH
50
37
Supplementary Fig. 7A
PDI
75
50
37
TUBULIN
75
50
37
MTCO2
75
50
37
Supplementary Fig. 7B
LACC1
75
50
37
25
GAPDH
75
50
37
25
Supplementary Fig. 10A
RIP2
75
50
GAPDH
50
37
Supplementary Fig. 13A
LACC1
75
50
37
25
GAPDH
75
50
37
25
Supplementary Fig. 14A
LACC1
75
50
37
25
GAPDH
75
50
37
25
Supplementary Fig. 14B
SDHA
75
50
GAPDH
50
37
Supplementary Fig. 15A
IB:p-Tyr
50
37
WCL: LACC1
50
37
WCL: GAPDH
50
37
Supplementary Fig. 15C
IB:acetyl-Lys
75
50
25
WCL: LACC1
50
37
WCL: GAPDH
50
37
Supplementary Fig. 16A
IB:RIP2
75
50
37
WCL: LACC1
50
37
IB:IRAK1
75
50
37
WCL: GAPDH
37
25
Supplementary Fig. 18
IB:LACC1
75
50
37
IB:RIP2
75
50
37
IB:IRAK1
75
50
37
WCL: LACC1
75
50
37
IB:TRAF6
75
50
37
WCL: GAPDH
75
50
37

Supplementary Table 1. Primer sequences

Gene	Primer Sequence
LACC1	FWD: 5' CCGCCCTATCCCTCCTCAAG 3'<br>REV: 5' GCGTTAGAGGAACAGGAACCA 3'
GAPDH	FWD: 5' GAACGGTGTCTCCTTCACTTC 3'<br>REV: 5' TGCACCACCAACTGCTTAGC 3'
TRAF1	FWD: 5' TAAAGTGGTGGCACCTGACC 3'<br>REV: 5' ACCCTATCTTGCCTGGGACT 3'
MARCSL1	FWD: 5' GGCTACAGAGCCATCCACTC 3'<br>REV: 5' TGACCTCACAAGGACAGCAC 3'
CSF2	FWD: 5' GGGAGCATGTGAATGCCATC 3'<br>REV: 5' GGCTCCTGGAGGTCAAACAT 3'
ICAM1	FWD: 5' TCTTCCTCGGCCTTCCCATA 3'<br>REV: 5' AGGTACCATGGCCCCAAATG 3'
A20	FWD: 5' CAGAAGAAACTCAACTGGTGTCG 3'<br>REV: 5' GCCGTCACCGTTCGTTTT 3'
IL1A	FWD: 5' CTTCTGGGAAACTCACGGCA 3'<br>REV: 5' AGCACACCCAGTAGTCTTGC 3'
IRG1	FWD: 5' GATCAACAGGCCACAGGTGA 3'<br>REV: 5' TTCCTCTAGGTCCTCCTGGC 3'
RASL11A	FWD: 5' AGCATGTCCGGGCACTTTC 3'<br>REV: 5' GCACGATCATTGCGCTCTTG 3'
TLR2	FWD: 5' GGCCAGCAATTACCTGTGTG 3'<br>REV: 5' AGGCGGACATCCTGAAACCT 3'
CDC42EP2	FWD: 5' CCTTGGGCCAACTTTGTTCG 3'<br>REV: 5' CAGCCAGAGGAAACACTCGT 3'
IL6	FWD: 5' CACCATCCTGAGGGAAGAGG 3'<br>REV: 5' CGTCGGCACCCAAGAATT 3'
FSCN1	FWD: 5' AATCAGGACGAGGAGACCGA 3'<br>REV: 5' TCACGCCACTCGATGTCAAA 3'
SLC7A2	FWD: 5' AATATGTCGTCGCAGCTGGT 3'<br>REV: 5' CTGCCTCTTACTCACTCTGGC 3'
NKX3-1	FWD: 5' CCGAGCCAGAAAGGCACTTG 3'<br>REV: 5' CTTAGGGGTTTGGGGAAGCC 3'
GPR132	FWD: 5' GCCGCTGCCTTTTCCTACTA 3'<br>REV: 5' CGTGGCCAGCACGTAGATAA 3'
OLR1	FWD: 5' GCGACTCTAGGGGTCCTTTG 3'<br>REV: 5' CCTGGGATAATTGCATGCCC 3'
SOCS3	FWD: 5' ACCTGAACTCGCACCTCCTAC 3'<br>REV: 5' CAACCCCTGGTTTGTGCA 3'
IL1B	FWD: 5' ACAGATGAAGTGCTCCTTCCA 3'<br>REV: 5' GTCGGAGATTCGTAGCTGGAT 3'
MMP14	FWD: 5' CGCTGCCATGCAGAAGTTTT 3'<br>REV: 5' TCATGGCCTTCATGGTGTCT 3'
SLC27A4	FWD: 5' ATTGGTGAACTGTGCCGCTA 3'<br>REV: 5' GAAGTTGCCCAGGCTACAGT 3'

CD83	FWD: 5’ GAACGCGCGGGCATAAAAG 3’<br>REV: 5’ AGGGCAAGTCCACATCTTCG 3’
SLC1A2	FWD: 5’ GGTGATGTCAGCTCTCGACG 3’<br>REV: 5’ CAGGGAGGGATTGCAAGGTT 3’
S100A3	FWD: 5’ CTTCAAGGACTGCCCCTCAG 3’<br>REV: 5’ CTGATCGCAGAAGACCTGGG 3’
MMP11	FWD: 5’ GATCGACTTCGCCAGGTACT 3’<br>REV: 5’ CCCCGATAGTCCAGGTCTCA 3’
STX11	FWD: 5’ TCCCAGTCCAGGCAAAATGA 3’<br>REV: 5’ TGGGGCGAGTCAAACTCATC 3’
CD69	FWD: 5’ GAGCTGGACTTCAGCCCAAA 3’<br>REV: 5’ CCACTTCCATGGGTGACCAG 3’
IFIT1	FWD: 5’ CTGGCTAAGCAAAACCCTGC 3’<br>REV: 5’ AGCCTATGGAGGAAGGCTGT 3’
CD40	FWD: 5’ GCTGCTGAATGATGGGTATGG 3’<br>REV: 5’ GTCACCACTCTTCGAGCTGT 3’
MMP19	FWD: 5’ TCGCCTCGAACACAATGGAT 3’<br>REV: 5’ CTTCTTGGGGAAGCCAGGAG 3’
ICOSL	FWD: 5’ CGTGTACTGGATCAATAAGACGG 3’<br>REV: 5’ TGAGCTCCGGTCAAACGTGGCC 3’
CKB	FWD: 5’ TCATCGAGATGGAACAGCGG 3’<br>REV: 5’ CCAAGGGTGACGGAAGTCTC 3’
SLC2A6	FWD: 5’ CCTGCAGTCCATCTTCGACA 3’<br>REV: 5’ AAACATGATGGCCGCTGAGA 3’
TNF	FWD: 5’ AGCCTCTTCTCCTTCCTGATCGTG 3’<br>REV: 5’ GGCTGATTAGAGAGAGGTCCCTGG 3’
GPR84	FWD: 5’ AGGACTGCTCTTTGGGTGAG 3’<br>REV: 5’ GTTCCACATGATAGAGGCTGAGT 3’
CXCL1	FWD: 5’ TTGAGTCCCAACAGTCCACC 3’<br>REV: 5’ CTGGCTTAGAACAAAGGGGCT 3’
MMP12	FWD: 5’ AACCAACGCTTGCCAAATCC 3’<br>REV: 5’ TTTCCCACGGTAGTGACAGC 3’
CXCL10	FWD: 5’ AGCAGAGGAACCTCCAGTCT 3’<br>REV: 5’ ATGCAGGTACAGCGTACAGT 3’
RELB	FWD: 5’ GCTCTACTTGCTCTGCGACA 3’<br>REV: 5’ GGCCTGGGAGAAGTCAGC 3’
MMP10	FWD: 5’ CCTGGGTTTTCCTCCAACCA 3’<br>REV: 5’ TCTAGGGAAGCCTTGCTCCA 3’
CCL5	FWD: 5’ GACACCACACCCTGCTGCT3’<br>REV: 5’ TACTCCTTGATGTGGGCACG 3’
NFKB2	FWD: 5’ ACACCGTTGTACAAAGATACGC 3’<br>REV: 5’ GCCCGGCTCTGTCTAGTG 3’
NFKBIZ	FWD: 5’ CCTGGGAGCATGATTGTGGA 3’<br>REV: 5’ AAGTAGCTCAGGTTGAGCGG 3’

CXCL2	FWD: 5’ CGTTCTCGGAGAGCCACAG 3’<br>REV: 5’ AGGGGCGCTCCTGCT 3’
NFKBIE	FWD: 5’ TGGGCATCTCATCCACTCTG 3’<br>REV: 5’ TCTCTCTGCTATGGGTGTGC 3’
TNFAIP3	FWD: 5’ GCATACAACTGAAACGGGGC 3’<br>REV: 5’ GGGGTGTGATCTCTCTTGGC 3’
REL	FWD: 5’ CGAACCCAATTTATGACAACCG 3’<br>REV: 5’ TTTTGTTTCTTTGCTTTATTGCCG 3’
CXCL11	FWD: 5’ GTTCAAGGCTTCCCCATGTTC 3’<br>REV: 5’ ATAAGCCTTGCTTGCTTCGAT 3’
PDE4B	FWD: 5’ CGAGGGCAGCCTGAGGTATT 3’<br>REV: 5’ CCGTCATCACACTCCTGCTT 3’
CSF1	FWD: 5’ GGCGTTGGGCACTATCCAAG 3’<br>REV: 5’ ATTCCGACTATGCCCGGCTC 3’
EGR1	FWD: 5’ CTGACCGCAGAGTCTTTTCCT 3’<br>REV: 5’ GAGTGGTTTGGCTGGGGTAA 3’
MCLON3	FWD: 5’ GCTCTCCTCCCGTCTGACTCT 3’<br>REV: 5’ CTGCAGCTACTCACAACTACCT 3’
EGR2	FWD: 5’ TTGGAAGTGTCCTTGGTCGC 3’<br>REV: 5’ AGGCAACCCATTTACATGCAG 3’
MPO	FWD: 5’ TGGGGGTTCCCTTCTTCTCT 3’<br>REV: 5’ AGGACAGCTGGAGCAGCA 3’
PPAP2B	FWD: 5’ AGGATTTGCTCAAGGAGCCC 3’<br>REV: 5’ AGGGAGAGCGTCGTCTTAGT 3’
PRDM1	FWD: 5’ AGCCAGACGGTTAACACAGA 3’<br>REV: 5’ CTCTTGCCTCTCCGCAACA 3’
HSPA1A	FWD: 5’ GAGCAGGTGTGTAACCCCAT 3’<br>REV: 5’ TGAAGCTCCAAAACAAAAACAGC 3’
EGR3	FWD: 5’ CCGGTGACCATGAGCAGTTT 3’<br>REV: 5’ TCGTTGGTCAGACCGATGTC 3’
THBS1	FWD: 5’ CCATGCTTATTTGTTCTCTACTGGC 3’<br>REV: 5’ TAAGCCTAGGCCTGAGCAAC 3’
F5	FWD: 5’ CTCCGGGCTGTCCCAG 3’<br>REV: 5’ TAGAACTGCCTTAGCTGTGCC 3’
FABP4	FWD: 5’ TGGGCCAGGAATTTGACGAA 3’<br>REV: 5’ CACATGTACCAGGACACCCC 3’
ADAMTS1	FWD: 5’ CTCCAACTTTGCGCTGGAAG 3’<br>REV: 5’ AGCTCCCGGAGTCACTAAGA 3’
HSPA1B	FWD: 5’ GAGCAGGTGTGTAACCCCAT 3’<br>REV: 5’ ACAGCAGCAAAGTCCTTGAGT 3’
VDAC3	FWD: 5’ GAACGGTGTCTCCTTCACTTC 3’<br>REV: 5’ TGGAGGAATACACATTGGACG 3’
SDHA	FWD: 5’ GAAGCCCTTTGAGGAGCACT 3’<br>REV: 5’ TCCAGAGTGACCTTCCCAGT 3’
